# Epigenetics of Malignant Melanoma: Mechanisms, Diagnostic Approaches and Therapeutic Applications

**DOI:** 10.32604/or.2026.073894

**Published:** 2026-03-23

**Authors:** Sophiette G. Hong, George F. Murphy, Christine G. Lian

**Affiliations:** 1 Department of Human Developmental and Regenerative Biology, Harvard University, Cambridge, MA 02138, USA; 2 Program in Dermatopathology, Department of Pathology, Brigham and Women’s Hospital, Mass General Brigham, Harvard Medical School, Boston, MA 02115, USA

**Keywords:** Epigenetics, malignant melanoma, DNA/RNA modification, histone modification, chromatin remodeling

## Abstract

Malignant melanoma (MM) is a highly aggressive skin cancer known for its rapid progression, potential for metastasis, and resistance to treatment. Despite advances in targeted therapies and immunotherapy, the prognosis for metastatic melanoma remains unfavorable. Recent research has shed light on the significance of epigenetic modifications in the pathogenesis of melanoma, revealing critical mechanisms of melanoma development and progression. Epigenetic modifications, including DNA and RNA modifications, histone modifications, chromatin remodeling, and non-coding RNA regulation, disrupt normal gene expression without modifying the DNA sequence, leading to cellular transformation, invasion, immune evasion, and therapeutic resistance. The reversible nature of epigenetic modifications opens up new opportunities for melanoma recognition and classification, as well as therapeutic applications, including the development of diagnostic and prognostic biomarkers and innovative targeted therapies aimed at restoring normal gene function and enhancing the efficacy of existing treatments. This review will focus on the multifaceted role of epigenetic dysregulation in melanoma. The future integration of epigenetic data and genomic profiling with clinical outcomes, likely facilitated by artificial intelligence (AI) algorithms, holds promise for personalized treatment strategies that are informed by precise and combinatorial diagnostic tools, ultimately improving melanoma care. The study aims to deliver a comprehensive overview of the current state of epigenetics in melanoma.

## 1 Epigenetic Mechanisms in Melanoma Pathogenesis

Epigenetics involves modifications in gene expression that may be heritable but are without DNA sequence alterations [1]. The mechanisms of epigenetic aberration include DNA [2], histone [3], and RNA modification [4]; chromatin remodeling [5]; and non-coding RNA regulation [6]. The proteins involved in epigenetic modifications have recently been functionally categorized as writers, erasers, readers, and remodelers [7,8]. Writers and erasers are enzymes that have opposite effects on gene expression, as erasers undo what writers have modified on DNA bases or amino acids. Readers are protein domains that bind to DNA modification sites to recruit other enzymes for gene regulation. Remodelers affect chromatin by modifying chromatin accessibility via attaching or detaching nucleosomes at enhancer and promoter sites [9].

**Figure 1:**
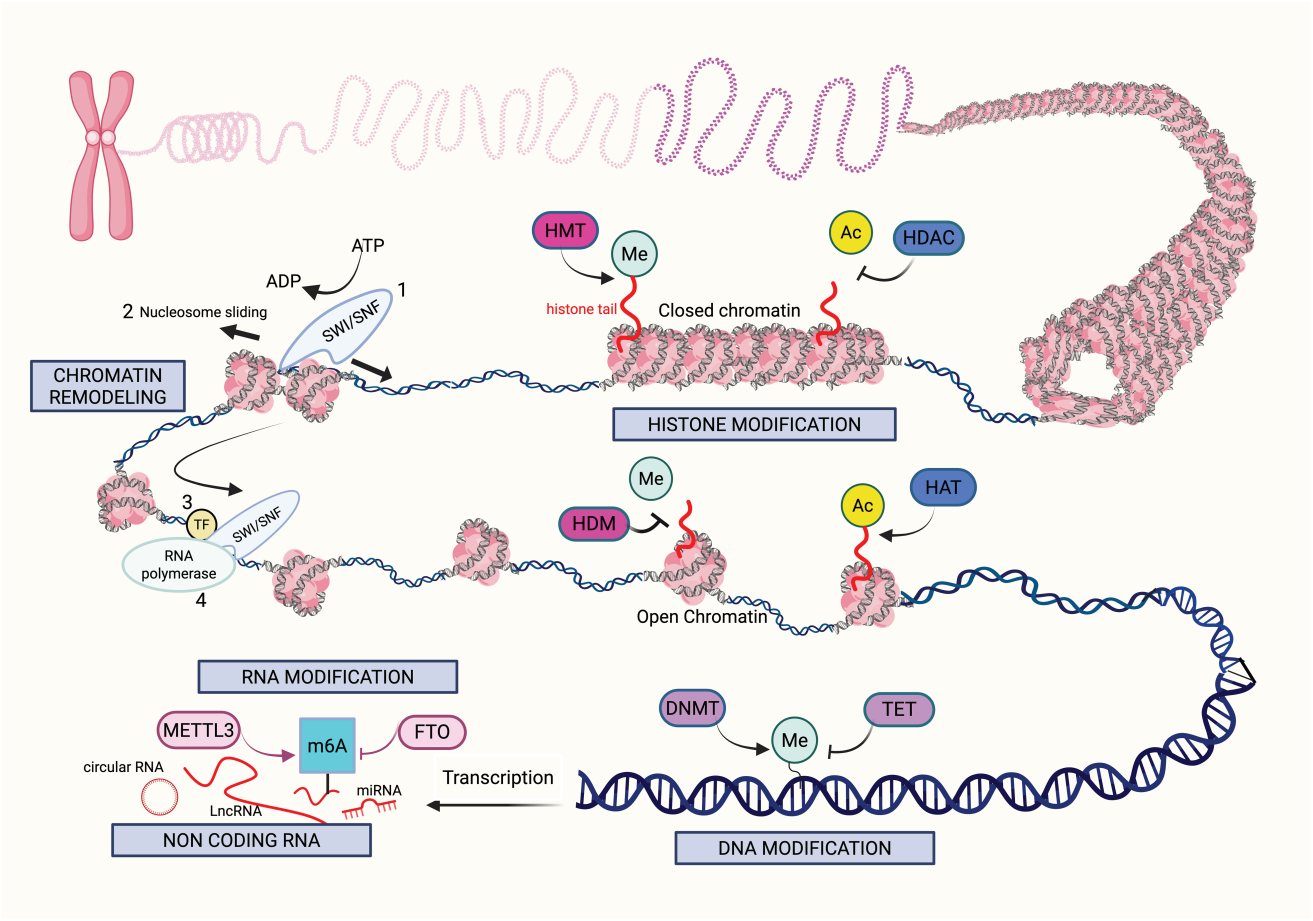
Mechanisms of epigenetic modifications in melanoma. The figure provides an overview of the key mechanisms (gray boxes) that drive epigenetic alterations in melanoma, including histone modification, chromatin remodeling, DNA modification, RNA modification, and noncoding RNA. Histone modifications, such as methylation (Me) and acetylation (Ac), are performed by “writers” like histone methyltransferases (HMT) and histone acetyltransferases (HAT). In contrast, histone deacetylases (HDAC) and histone demethylases (HDM) function as “erasers,” removing these modifications. Histone methylation can be associated with both open and closed chromatin structures, and histone acetylation typically promotes a more open chromatin structure, allowing greater access for transcription factors. Chromatin remodeling initiates with SWItch/Sucrose Non-Fermenting (SWI/SNF) complexes, disrupting histone-DNA interactions by mobilizing nucleosomes, thereby increasing DNA accessibility, which facilitates the binding of transcription factors (TFs) and RNA polymerase to promoter and enhancer regions of the DNA. DNA modifications are mediated by “writers” such as DNA methyltransferases (DNMT) and “erasers” such as Ten-Eleven Translocation (TET) enzymes. An example of RNA modification includes Methyltransferase-like 3 (METTL3), which is a N6-methyladenosine (m6A) “writer”, as it adds methyl groups to adenosine bases on RNA, specifically m6A. Demethylation by Fat mass and obesity-associated protein (FTO) is the “eraser” of this reaction, removing the m6A from the RNA. Lastly, noncoding RNAs such as long noncoding RNAs and microRNAs possess complex epigenetic functions that can alter melanoma pathogenesis. *Created in BioRender. Hong, S. (2025)*

In melanoma, these epigenetic processes are frequently dysregulated and have been recognized as critical factors in cancer development, metastasis, and treatment resistance [10]. The reversibility of the enzymes that catalyze the epigenetic reactions opens up vast possibilities for innovation in drug targeting. We will discuss the epigenetic mechanisms of melanoma pathogenesis in the following order: DNA modification, histone modification, non-coding RNA regulation, chromatin remodeling, and RNA modification. Fig. 1 illustrates the overview of the key mechanisms of epigenetic modifications in melanoma.

### 1.1 DNA Modification in Melanoma

DNA methylation involves the covalent transfer of a methyl group to the 5^′^ position of cytosine, producing 5-methylcytosine (5mC). Methylation most commonly occurs at the Cytosine-phosphodiester bond-Guanine (CpG) dinucleotides concentrated at the gene promoter region, thus forming “CG islands” [11]. The writer enzymes in DNA methylation are DNA methyltransferases (DNMTs) [12,13], and the eraser enzymes are the ten-eleven translocation (TET) methylcytosine dioxygenases. Hydroxymethylation of 5mC converts it into an intermediary, 5-hydroxymethylcytosine (5-hmC) [14,15], which is interestingly known to be present at elevated levels in self-renewing and pluripotent embryonic stem cells [15,16].

One common mechanism of aberrant DNA methylation in the development of cancer involves hypermethylation of CG islands (CGIs) in gene promoters and global loss of DNA methylation. DNA hypermethylation of tumor suppressor and DNA repair genes impairs the proliferation and differentiation of normal cells, while promoting the development of cancer cells [17–20]. Global loss of DNA methylation causes chromosomal instability [21,22]. Additionally, specific loss of DNA methylation in oncogene promoter regions will also promote malignancy [23]. Various mechanisms of altered DNA methylation play complex roles in cancer development and progression in melanoma, contributing to the loss of cell cycle regulation, apoptosis, proliferation, cell invasion, and metastasis [24]. Global hypomethylation of DNA is a key feature of cancer, marked by a general reduction in 5-methylcytosine throughout the genome [25,26], which encompasses the intergenic region and is present within the introns of DNA, especially in repetitive sequences and transposable elements [27]. Some of these globally hypomethylated repetitive sequences are LINE1, *Alu*, and Satα [28,29]. Global DNA hypomethylation has been linked to the initial phases of cancer development in diverse cancer types [30,31]. There are indications that hypomethylation may play a role in cancer development by activating oncogenes and causing chromosome instability since methylation of pericentromeric areas is essential for maintaining chromosome stability [23].

In malignant melanomas, global hypomethylation has been observed [32]. Compared to nevi, a decrease in 5mC levels is noted in melanomas, which indicates that DNA hypomethylation may have a significant role in the survival of melanoma cells [33]. In addition to global changes, hypomethylation of specific regions/genes has been studied. Hypomethylation activates certain Cancer Testis (CT) genes [34–36], including Melanoma Antigen Genes (MAGE) [37], B Melanoma Antigen (BAGE), G Antigen (GAGE), and New York-Esophageal Squamous cell carcinoma-1 (NY-ESO-1) [38,39]. Other genes with regional hypomethylation include the feline sarcoma (FES) [40], Deleted split-hand/split foot 1 (DSS1) [41], Carnosine dipeptidase 1 (CNDP1) [42], and Tre-2/Bub2/Cdc16-1 Domain Family Member 16 (TBC1D16) [43].

We reported that loss of 5-hmC is an epigenetic hallmark with important functional, diagnostic, and prognostic implications in melanoma [44]. We found that decreased activity of Isocitrate Dehydrogenase 2 (IDH2) and TET family enzymes represents critical mechanisms responsible for the reduction of 5-hmC in melanoma [44]. Of interest, alterations in the genes IDH1 and IDH2 in cancer cells lead to the formation of the oncometabolite 2-hydroxyglutarate (2-HG) [45]. This compound acts as an antagonist to α-ketoglutarate (α-KG), which is essential for the conversion of 5mC to 5-hmC [46]. In animal models, we learned that reintroducing active TET2 or IDH2 in melanoma cells was effective in reducing melanoma growth and extending tumor-free survival. This research highlights the crucial role of 5-hmC in the development and virulence of melanoma, establishing a direct connection between the IDH and TET activity-dependent epigenetic mechanisms and the suppression of melanoma progression through 5-hmC [44]. Moreover, we and others have also found loss of 5-hmC 1) in fields of presumed precursor melanocytes in which melanomas arose [47–50]; 2) in dysplastic nevus precursors in which some melanomas developed [51]; 3) in association with melanoma progression and metastatic process [33,48,52,53]. Epigenetic modifications including the progressive loss of 5-hmC are considered a key component of melanocyte field cancerization [49,50].

We recently examined the expression of Preferentially Expressed Antigen in Melanoma (PRAME) and found that 5-hmC, in conjunction with the activation of PRAME, contributes significantly to the transformation and progression of melanoma. The study revealed that TET2 is significantly less active in melanoma compared to melanocytes found in normal skin, and that this lower activity in melanoma is associated with decreased levels of 5-hmC and increased expression of PRAME [54]. Notably, levels of 5-hmC were reduced at the PRAME 5^′^ promoter in melanoma compared with nevi, suggesting a role for 5-hmC in PRAME transcription. Restoration of 5-hmC levels via TET2 overexpression in melanoma cell lines markedly reduced PRAME expression, thus establishing a function of TET2-mediated DNA hydroxymethylation in regulating PRAME expression and demonstrating that epigenetic reprogramming plays a potentially pivotal role in melanoma tumorigenesis.

Since Horvath first introduced the concept of universal epigenetic clock for assessing biological age, subsequent research has shown that cancers often exhibit DNA methylation patterns that indicate they are biologically older than healthy tissue, a phenomenon known as “age acceleration” [55]. Aberrant DNA methylation in melanoma dysregulates various cellular processes, including cell cycle, cell signaling, transcription, DNA repair, and apoptosis [35,56–58]. DNA methylation is also found to be associated with the progression of metastatic melanoma. When the metastasis driver, Nuclear Receptor Subfamily 2 Group F, Member 2-isoform 2 (NR2F2-Iso2) is hypomethylated and re-expressed, it enables melanocytes to acquire features similar to those of neural crest cells during metastasis [59]. Table A1 categorizes the genes with aberrant DNA methylation based on the predominant pathogenic mechanism(s) in melanoma evolution and progression.

Phosphatase and TENsin homolog (PTEN) acts as a lipid phosphatase that converts phosphatidylinositol (3,4,5)-triphosphate (PIP3) to PIP2, thereby directly antagonizing phosphatidylinositol 3-kinase (PI3K) signaling. PI3K activation drives hyperactivation of the Ak strain transforming/mammalian target of rapamycin (AKT/mTOR) pathway [60]. This hyperactivation promotes cell proliferation, survival, invasion, and immune evasion through mechanisms such as enhanced Fos-related antigen 1 (FRA1) translation, inactivation of Forkhead box O (FOXO), and increased Programmed Death-Ligand 1 (PD-L1) expression [61]. Hypermethylation has been found to be a significant mechanism of PTEN loss in 60% of melanomas in one study [62,63], although another study found that methylation is less critical, as PTEN is often inactivated through DNA mutations or deletions. However, when PTEN mRNA expression and methylation were plotted and compared, 97.9% of melanomas showed less than 10% PTEN methylation [64]. Cyclin-Dependent Kinase Inhibitor 2A (CDKN2A) encodes two tumor suppressors: protein16 inhibitor of CDK4/6 (p16INK4a), which inhibits CDK4/6 to prevent retinoblastoma tumor suppressor gene (RB) phosphorylation and cell cycle progression, and p14 Alternate Reading Frame (p14ARF), which stabilizes p53 by sequestering murine double minute 2 (MDM2). Loss of CDKN2A through deletion, mutation, or methylation results in uncontrolled E2F activity, directly activating the Brain-2 (BRN2) invasion program and simultaneously eliminating both RB and p53 tumor suppressor checkpoints [65]. Methylation of the CDKN2A gene in melanoma is a key example of a writer alteration in DNA methylation. The methylation leads to the loss of its tumor-suppressive functions, promoting the development of melanoma, and correlating with a poor prognosis. The CDKN2A p16 is hypermethylated in certain cutaneous melanomas, leading to cell cycle arrest at the G1-S checkpoint by inhibiting the proteins CDK4/6 [66–69]. P14ARF (CDKN2A) is found to be hypermethylated in cutaneous and uveal melanomas. One study of melanomas in the vertical growth phase showed that 19% of the cases had CDKN2A promoter region hypermethylation, and interestingly, some instances were heterogeneous with tumor cells that were both methylated and unmethylated [65,68,70]. Ras association domain family 1 isoform A (RASSF1A) may exhibit hypermethylation at its promoter regions. This hypermethylation has been identified in 55% of melanoma tumors, while normal skin shows no detectable methylation. The hypermethylation leads to a halt in the cell cycle (G1 to S phase) and increases the expression of ASK1 (Apoptosis signal-regulating kinase 1), a protein kinase involved in stress-induced cellular responses, particularly apoptosis (programmed cell death) and inflammation. The extent of RASSF1A methylation differs according to the stage of the tumor, and its reduced expression inhibits apoptosis [71,72].

Telomerase Reverse Transcriptase (TERT) promoter-activating mutations were initially identified at a high rate in cutaneous melanoma [73,74], and subsequent studies have indicated their presence in various other cancers [75,76]. Upregulation of TERT plays a role in maintaining telomeres, which is vital for cellular immortality and the survival of cancer cells. While TERT promoter mutations (TPMs) significantly contribute to TERT upregulation in cancer, many tumors show TERT upregulation without these mutations. Research has identified the TERT hypermethylated oncological region (THOR), situated just upstream of the TERT core promoter, as an epigenetic site associated with TERT upregulation in cancer. When THOR is unmethylated, it inhibits TERT promoter activity, regardless of the presence of TPM, while hypermethylation of THOR reverses this inhibitory effect. Thus, THOR hypermethylation is suggested to be a common mechanism for activating telomerase in cancer that can work independently or in collaboration with TPMs [77]. However, the effect of TERT epigenetic regulation on melanoma progression has shown discrepant findings. A study of normal skin samples and 61 melanoma cell lines was conducted to clarify the effects of epigenetic and genetic mechanisms in regulating TERT gene expression. TERT gene expression requires a high promoter methylation level, open chromatin, and the absence of mutations. The TERT gene is also expressed with a moderately methylated promoter and existing mutations. Thus, there is a complex interplay between the promoter methylation, chromatin accessibility, and promoter mutation status [78]. TERT promoter methylation was found to indicate worse prognosis in young melanoma patients, as the promoter methylation alone or combined with promoter mutations correlated with reduced recurrence-free survival, whereas only having the TERT promoter mutation did not correlate with prognosis [79]. O6-methylguanine-DNA methyltransferase (MGMT) is an enzyme that repairs the impact of methylation on DNA, a process that removes alkyl groups from the O6 position of guanine. In melanoma, it is common to find hypermethylation of the MGMT promoter, leading to the silencing of the MGMT gene. This silencing renders the melanoma cells more susceptible to specific chemotherapeutic drugs that utilize alkylating agents to induce DNA damage, leading to improved treatment outcomes. Importantly, the methylation status of MGMT may also serve as a prognostic marker, influencing treatment choices [80,81].

### 1.2 Histone Modification in Melanoma

Chromatin consists of building blocks of nucleosomes, which consist of DNA (146 base pairs) organized around a histone protein octamer (2 copies of H2A, H2B, H3, H4) [82,83]. Histone modification is a dynamic process influenced by specific enzymes that alter charges determined by nucleosomal structure. This modification either strengthens or weakens interactions between histones and DNA that regulate transcriptional activation and repression [84,85]. Histone modifications primarily occur on the lysine-rich N-terminal tails [86] and cause abnormalities in chromatin structure, influencing gene expression related to cell differentiation, proliferation, and survival. Abnormal histone modifications such as acetylation, phosphorylation, methylation, and ubiquitination significantly contribute to melanoma development by activating oncogenes and silencing tumor suppressor genes. The most common types of epigenetic modifications in melanoma involve the histone acetyl and methyl groups.

#### 1.2.1 Histone Acetylation and Deacetylation

Histone Acetyltransferases (HATs): Writers

The positive charge of histone tails and the negative charge of DNA form a tight bond inherent in the closed heterochromatin structure, and HATs neutralize this histone positive charge to increase chromatin accessibility, thus enhancing transcription [87,88]. Protein 300/CREB-binding protein (P300/CBP) is a biomarker protein produced by histone 3 at lysine 27 (H3K27Ac) acetylation that is neutralized by HAT. Protein 300 and CBP are HATs vital in regulating chromatin dynamics and gene expression. They are involved in various cellular functions, including proliferation, differentiation, and immune responses. Disruption of p300/CBP function has been associated with the onset and progression of melanoma, which includes activating oncogenic transcription factors such as Microphthalmia-Associated Transcription Factor (MITF) and Sex-determining region Y (SRY)-related High-Mobility group (HMG) box (SOX)10, as well as modulating cell cycle progression. Indeed, inhibiting p300/CBP can decrease melanoma cell proliferation and alter gene expression related to melanoma development [89–92].

Bromodomain and Extra-Terminal Domain (BET) Proteins: Readers

BET proteins, specifically Bromodomain-Containing Protein 2 (BRD2) and BRD4, function as readers, and they are both upregulated by acetylation of lysine residues of histones. In melanoma, these proteins are often up-regulated and play a role in tumorigenesis by modulating the expression of essential cell cycle and survival genes. When BRD2 and BRD4 are displaced from chromatin, transcription is inhibited, leading to the deactivation of cell cycle genes such as Extracellular signal-Regulated Kinase 1 (ERK1), cellular myelocytomatosis oncogene (c-MYC), and S-phase kinase-interacting protein 2 (SPK2). This results in G1/S phase arrest and cell death [93]. Segura et al. found that BRD4 levels are elevated in both primary and metastatic melanoma compared to normal melanocytes and melanocytic nevi. The use of BET inhibitors has been shown to hinder melanoma cell growth *in vitro* as well as impede tumor growth and metastatic activity *in vivo*. Notably, the effectiveness of these inhibitors is not dependent on the mutational status of B-Raf proto-oncogene, serine/threonine kinase (BRAF) or neuroblastoma RAS viral oncogene homolog (NRAS), suggesting that these small-molecule therapies could represent viable treatment options [94].

Histone Deacetylases (HDACs): Erasers

HDACs—such as HDAC6 [95,96], HDAC1 [97], HDAC3 [98] and HDAC8 [98]—are erasers that reverse acetylation to form closed chromatin with decreased gene expression. In melanoma, HDAC activity is often upregulated, silencing tumor suppressor genes and activating the Mitogen-Activated Protein Kinase (MAPK) pathway. While HDACs silence tumor suppressors, their interaction with the MAPK pathway is bidirectional, involving feedback loops and resistance mechanisms rather than unidirectional activation. Immune evasion with HDAC upregulation has multiple pathways. Histone deacetylation mediated by HDACs suppresses the expression of Major Histocompatibility Complex (MHC) class I molecules and essential components of the antigen processing machinery, such as the proteasome subunits low molecular mass polypeptide (LMP)-2 and LMP-7 and the Transporter Associated with Antigen Processing (TAP) transporter. This suppression occurs through the formation of a more condensed chromatin structure, which inhibits transcription of these genes in melanoma and various other cancer types [99]. Treatment with HDAC inhibitors increases the expression of TAP1, TAP2, LMP2, LMP7, tapasin, and MHC class I molecules in melanoma cells. This upregulation leads to enhanced cell surface expression of class I molecules and costimulatory molecules CD40 and CD86, thereby promoting direct presentation of whole protein antigens and MHC class I-restricted peptides [100]. HDAC2 is recruited to the PD-L1 promoter by STAT1 and facilitates PD-L1 induction by increasing phosphorylation of Janus kinase (JAK)1, JAK2, and Signal Transducer and Activator of Transcription (STAT)1. This process also enhances STAT1 nuclear translocation and its recruitment to the PD-L1 promoter. Knockout of HDAC2 impairs IFN-γ-induced upregulation of H3K27 and H3K9 acetylation, as well as BRD4 recruitment at the PD-L1 promoter [101]. HDAC upregulation is also associated with metastasis. SNAIL directly interacts with the E-cadherin promoter and recruits HDAC1, HDAC2, and the co-repressor Sin3A to the CDH1 promoter to silence E-cadherin expression by deacetylation of histones H3 and H4, an effect that was abolished by HDAC inhibitor trichostatin A treatment [102]. The recruitment of HDACs to the CDH1 promoter is regulated by transcription factor Zinc Finger E-box-Binding Homeobox (ZEB)1, with the Snail/HDAC1/HDAC2 complex essential for enhancer of zeste homolog 2 (EZH2)-mediated repression of CDH1 [103]. HDAC10 suppresses expression of matrix metalloproteinases (MMP)2 and MMP9, genes critical for cancer cell invasion and metastasis, while HDAC11 inhibits migration and invasion of cancer cells by downregulating MMP3 expression [104]. HDAC8 activation in melanocytes and melanoma cells is triggered by various stresses, prompting the cells to adopt a neural crest-stem cell-like state characterized by increased invasiveness and a higher tendency to metastasize to the brain. HDAC8 accomplishes this by deacetylating and inactivating the enzyme EP300. This change enhances EP300’s interaction with Jun-driven genes, while reducing its activity at MITF-controlled genes. As a result, inhibiting EP300 further promotes melanoma cell invasion, stress resistance, and brain metastasis. Overall, HDAC8’s suppression of EP300 shifts gene expression patterns to favor melanoma progression and brain metastasis [105].

#### 1.2.2 Histone Methylation and Demethylation

Histone Methyltransferases (HMTs): writers

Histone methylation activates or represses gene expression associated with melanoma progression. Commonly occurring at lysine or arginine residues, histone methylation determines gene expression based on the site and number of methyl groups added [106,107]. One significant histone lysine methyltransferase (HKMTase, HMT) is the EZH2, the primary component of the polycomb-repressive complex 2 (PRC2). PRC2 induces the trimethylation of histone H3 at lysine 27 (H3K27me3), a mark of tumor suppressor gene silencing. In melanoma, overexpression of EZH2 has been found to cause a high proliferation rate and is associated with aggressive tumor subgroups. There is also accumulating evidence that EZH2 plays a role in the progression and metastasis of melanoma [108,109]. EZH2-mediated H3K27me3 at MHC class I antigen processing pathway (MHC-APP) loci reduces basal expression of these genes and inhibits their interferon (IFN)-γ-induced activation, enabling tumor cells to evade immune surveillance by effector T cells [110]. EZH2-mediated H3K27me3 and DNA methylation repress tumor production of Th1-type chemokines Chemokine (C-X-C motif) ligand (CXCL)9 and CXCL10, which are critical for recruiting effector T cells to the tumor microenvironment. EZH2 inhibits CXCL9 transcription by increasing H3K27me3 at its promoter, thereby impeding CD8+ T cell trafficking to immune-desert tumors [111]. Protein Arginine Methyltransferase (PRMT)1-mediated methylation of EZH2 at arginine 342 strengthens EZH2 binding to target gene promoters and increases H3K27me3 levels, which is necessary for EZH2 to promote the epithelial-to-mesenchymal transition (EMT) program and stimulate cancer cell migration [112].

Other abnormal HMT writer modifications, such as up-regulation of SET domain bifurcated 1 (SETDB1), up-regulation of Lysine methyltransferase 2D (KMT2D), and up-regulation of Euchromatic Histone Methyltransferase (EHMT), also play a role in controlling transcription, chromatin structure, cell differentiation, and melanoma progression. SETDB1 is related to H3K9me3, leading to tumor suppressor gene silencing. Further, H3K4me1 causes activation of thrombospondin-1 (THBS1), which accelerates melanoma initiation and is related to metastasis [93,113]. Orouji et al. found that the activation of thrombospondin-1 (THBS1), which is known to enhance invasiveness and metastasis formation in melanoma, is triggered by SETDB1. In addition to increasing H3K9me3 at a global genomic level, SETDB1 also modifies the methylation patterns and affects H3K4me1 levels upstream of the THBS1 gene at a specific site for transcriptional activation. Thus, SETDB1 may influence not only the distribution of H3K9me3 but also impact other epigenetic markers that control gene activation or suppression. Importantly, using a small molecule inhibitor targeting H3K9me-specific histone methyltransferase to inhibit the SETDB1 protein significantly reduced melanoma cell viability [114]. Additionally, SETDB1 facilitates melanoma cells’ evasion of the immune system by epigenetically silencing genes, particularly endogenous retroviruses. Cuellar et al. show that removing SETDB1 in human leukemia cell lines activates these repetitive elements, leading to increased double-stranded RNAs. When SETDB1 is highly expressed in melanoma cells, it reduces the tumor’s capacity to activate an immune response, promoting immune evasion and resistance to immune checkpoint blockade (ICB) therapies [115].

The Disruptor of telomeric silencing 1-like (DOT1L) gene, often deleted or mutated in human melanoma, exhibits specific mutations that reduce its methyltransferase activity, leading to decreased H3K79 methylation. This reduction affects DNA damage repair by impairing the recruitment of the Xeroderma Pigmentosum complementation group C (XPC) protein to sites of damage, a crucial step in nucleotide excision repair. The findings suggest that DOT1L plays a protective role in preventing melanoma development induced by UV radiation [116].

KMT2D is an HMT responsible for adding a methyl group to histone H3 at lysine 4 (H3K4me1), marking enhancer regions. In melanoma, silencing KMT2D leads to the inactivation of a subset of KMT2D-bound enhancers, which results in decreased H3K4me1 and H3K27ac levels. Silencing KMT2D also downregulates genes critical for cell migration, such as MFGE8 and RPL39L. This alteration promotes tumorigenesis by disrupting enhancer activity [117,118].

PRMT1 modifications enhance the expression of PRMT1 and elevate levels of activated leukocyte cell adhesion molecule (ALCAM) through arginine methylation of histones. This process may contribute to the growth and metastasis of melanomas [119]. PRMT5 is emerging as a target for various solid and hematologic cancers. Its overexpression or dysregulation has been detected in multiple cancer forms, including melanoma.

Histone Demethylases (HDMs): erasers

HDMs such as Jumonji AT-rich interactive domain 1B (JARID1B, KDM5B) are erasers, removing methyl groups from histone 3 at the lysine residue position 4 (H3K4), Jumonji domain-containing protein 3 demethylase (JMJD3) at the lysine residue position 27 (H3K27), and Lysine-specific histone demethylase 1A (LSD1, KDM1A) at the lysine residues at positions 4 and 9 (H3K4 and H3K9). JARID1B is a histone demethylase that has a multifaceted role in melanoma. Research indicates that melanoma cells exhibit increased levels of JARID1B, suggesting that this may occur early in the progression of disease and does not correlate with the invasive phase of melanoma [120]. Chauvistré et al. suggest that JARID1B plays a significant role in tumor growth, maintenance, survival, and treatment resistance. Their research indicates that melanoma cells with elevated JARID1B levels form a slow-cycling stem cell-like subpopulation that can enter a reversible “persister” state crucial for continuous tumor growth. Maintaining this slow-cycling state inhibits melanoma growth and cell invasion. However, these melanoma cells can leverage this state to withstand targeted or cytotoxic therapies. The researchers propose that this idea could be applied as a strategy to improve responses in residual disease following advanced cancer treatments [121]. JMJD3 is a histone demethylase associated with the progression and metastasis of melanoma. JMJD3 is involved in the activation of Nuclear Factor (NF)-κB and Bone Morphogenetic Protein (BMP) signaling pathways that facilitate melanoma development as well as alter the melanoma tumor microenvironment by enhancing angiogenesis and recruiting macrophages [122]. KDM1A, also known as LSD1, is a lysine demethylase that plays a role in several cancers—including melanoma—and is being investigated as a potential therapeutic target. KDM1A has been implicated in the development and progression of melanoma [123,124].

#### 1.2.3 Histone Phosphorylation and Ubiquitination

Histone phosphorylation is implicated in the development of melanoma. It can alter chromatin architecture and influence transcriptional activation, particularly during cellular division [10,125]. Additionally, the phosphorylation of histones H1, H2B, and H3 significantly affects DNA repair mechanisms and gene regulation [10,126].

Ubiquitination is a form of post-translational modification that tags proteins for degradation or modulation of their functions. Ubiquitination influences various cellular processes, including the cell cycle, apoptosis, and DNA repair. Dysregulation within the Ubiquitin-Proteasome System (UPS) is often observed in melanoma, leading to abnormal degradation or stabilization of proteins that can propel tumor growth. Ubiquitination affects B-Raf proto-oncogene, serine/threonine kinase (BRAF) and Mitogen-activated extracellular signal-regulated kinase (MEK) in the MAPK pathway, enhancing survival signaling by stabilizing AKT [127,128]. Specific E3 ligases, such as MDM2, Itchy protein (ITCH), and RING finger protein (RNF)125, regulate oncogenes or tumor suppressors, thereby influencing melanoma progression [129,130]. Deubiquitinases (DUBs), such as USP9X and USP13, stabilize anti-apoptotic proteins, including Myeloid cell leukemia-1 (MCL-1). Targeting the UPS may aid in sensitizing melanoma cells to existing therapies or overcome resistance. The specificity of ubiquitination makes it an attractive target for the development of targeted drugs in melanoma [131].

#### 1.2.4 Other Histone Modifications

Additional less common post-translational histone modifications identified in cancer research include lactylation, citrullination, crotonylation, succinylation, SUMOylation, propionylation, butyrylation, 2-hydroxyisobutyrylation, 2-hydroxybutyrylation, ADP-ribosylation, butolylation, hydroxylation and formylation [132–134]. Lactylation, crotonylation, butyrylation, succinylation, and 2-hydroxyisobutyrylation are types of acylation that bridge cellular metabolism with chromatin regulation. For instance, higher lactate levels in tumors can increase histone lactylation, which changes gene expression. In melanoma, researchers have begun identifying specific histone lactylation sites, such as H3K18la, which is related to poor prognosis [135]. Sirtuin 5 (SIRT5), an eraser of succinylation, is required for proliferation and survival in melanoma [136]. Modifications such as citrullination, SUMOylation, and glycosylation [93] serve as molecular links between cellular stress, DNA damage, immune signaling, and chromatin remodeling in melanoma. Citrullination is catalyzed by peptidylarginine deiminases (PADs), which convert arginine residues to citrulline in histones, thereby altering chromatin structure. SUMOylation, the addition of small ubiquitin-like modifier (SUMO) proteins to histones, is up-regulated in melanoma and supports tumor growth [137]. These histone modifications represent promising avenues as biomarkers providing insights into cellular metabolic states and new opportunities for combination therapies.

### 1.3 Non-Coding RNA Regulation in Melanoma

Non-coding RNAs (ncRNAs) consist of microRNAs (miRNAs) that are less than 200 bp, typically 21–25 bp single-stranded RNAs, and long non-coding RNAs (lncRNAs) that are more than 200 bp and may extend to over 100 kb.

#### 1.3.1 LncRNA Regulation

LncRNAs are involved in gene regulation in melanoma. For example, the lncRNA Survival Associated Mitochondrial Melanoma Specific Oncogenic Non-coding RNA (SAMMSON) gene is targeted by the transcription factor SOX10, with high expression levels in over 90% of human melanomas. Increasing SAMMSON enhances the clonogenic potential of melanoma cells, while its knockdown significantly reduces cell viability, independent of genetic mutations. Silencing SAMMSON disrupts essential mitochondrial functions, specifically in cancer cells [138]. The lncRNA Metastasis Associated Lung Adenocarcinoma Transcript 1 (MALAT1) gene has been shown to promote melanoma cell migration and invasion, as well as inhibit apoptosis. Homeobox (HOX) transcript antisense intergenic RNA (HOTAIR) is suggested to directly interact with histone-modifying enzymes, which alter chromatin structure in melanoma development. Lymph node metastasis in melanoma showed overexpression of HOTAIR. The lncRNA HOTAIR functions as a molecular sponge for the miR-200 family, resulting in miR-200 downregulation and facilitating cancer progression. This regulatory axis disrupts immune checkpoint regulation by inducing epithelial-to-mesenchymal transition (EMT), a process linked to the development of an immunosuppressive tumor microenvironment and resistance to immune checkpoint inhibitors. Consequently, targeting the HOTAIR-miR-200 interaction represents a promising therapeutic strategy to improve immune checkpoint inhibitor efficacy, which is an important therapeutic aspect in melanoma treatment [139,140].

Additional oncogenic lncRNAs, such as BRAF-activated ncRNA (BANCR) and Antisense Non-coding RNA in the INK4 Locus (ANRIL), are found to inhibit apoptosis, promote invasion, and facilitate metastasis in melanoma [141].

#### 1.3.2 MicroRNA Regulation

MicroRNAs modulate gene expression of approximately 60% of human genes by binding to the 3^′^ untranslated regions (3^′^UTR) or 5^′^UTR of target mRNAs [142–144]. The miRNAs display a complex function as one miRNA can regulate multiple mRNAs, and multiple miRNAs can target one mRNA [145]. They are essential in governing processes such as cell development, growth, differentiation, and maintenance of homeostasis in both normal and diseased cells [146,147].

Research has elucidated a critical role for tumor cell-secreted exosomes throughout different phases of tumor progression and metastasis. These exosomes carry biomolecules, including proteins, RNA, and lipids, from tumor cells to their surrounding environment [148]. Extracellular RNAs (exRNAs) including miRNAs, lncRNAs, and mRNAs are aberrantly expressed in melanoma. Researchers have identified new metabolic reprogramming pathways and therapeutic targets such as the NEAT1-macrophage axis. ExRNAs contribute to melanoma progression by regulating the expression of target genes and mediating key signaling pathways. Melanoma-derived exRNAs reshape the tumor microenvironment. Exosomes containing miR-155 and miR-210 significantly reprogram the metabolism of fibroblasts, resulting in marked reductions in basal and maximal respiration, as well as ATP production, thereby creating a microenvironment that supports metastasis. While exRNA profiles show promise for real-time treatment adaptation and early recurrence detection, more research is needed before miRNAs, mRNAs, and proteins in extracellular vesicles become reliable cancer biomarkers [149]. Translational research has also identified numerous exosomal miRNAs in melanoma that aid tumor evasion of immune response [150].

**Figure 2:**
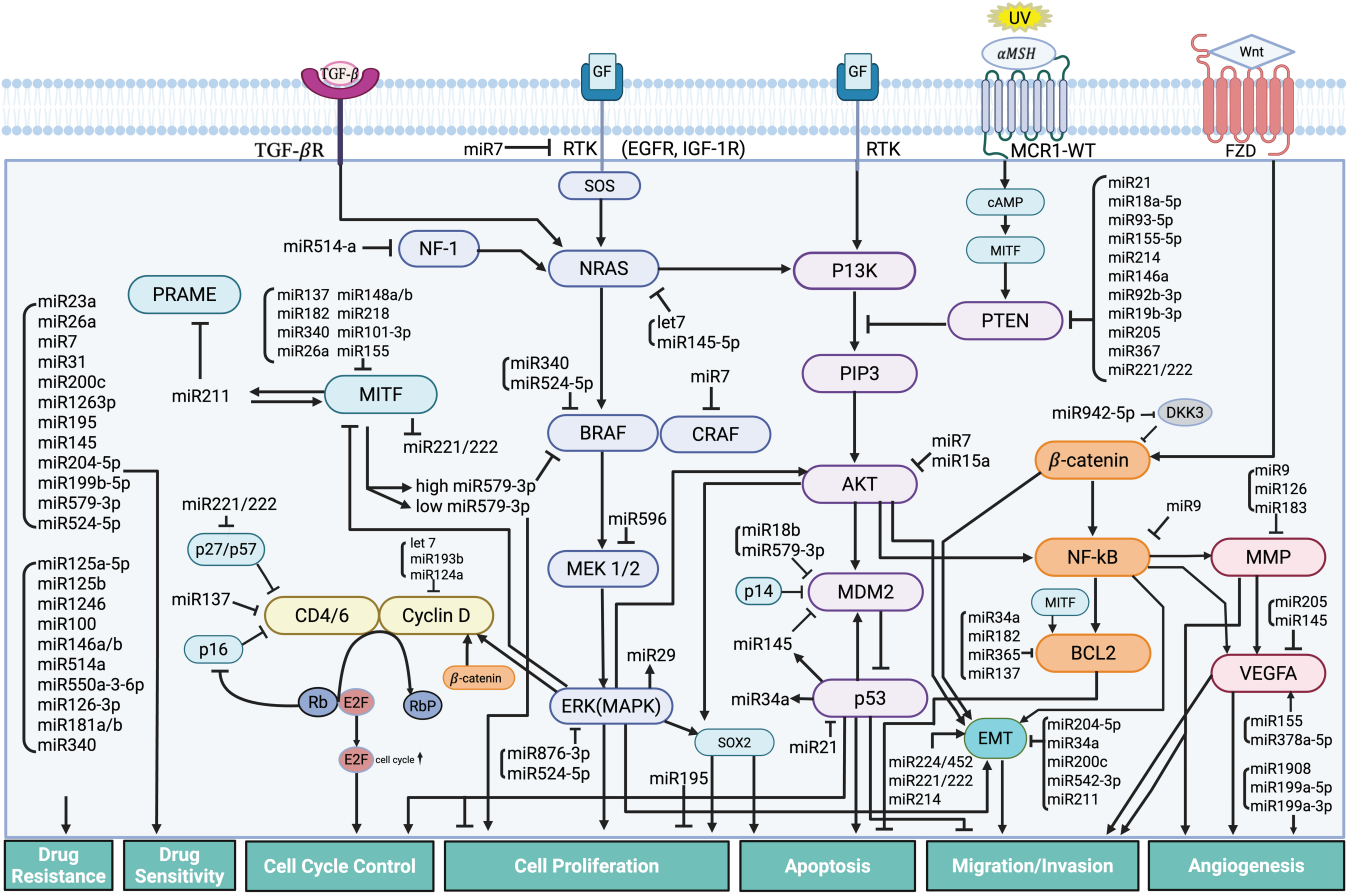
Mechanisms of MicroRNA Regulation in Cellular Pathways of Melanoma. The figure highlights the intricate network of miRNAs in regulating key cellular pathways involved in the development and progression of melanoma. miRNAs modulate multiple signaling cascades that influence critical processes such as cell proliferation, growth, and survival; cell cycle control; cell migration and invasion; apoptosis; drug resistance; and angiogenesis. Abb: PRAME, Preferentially expressed antigen in melanoma; SOX, SRY-box transcription factor; MITF, Microphthalmia-associated transcription factor; CDK, Cyclin-dependent kinase; NF-1, Neurofibromin-1; CDKN2A, Cyclin-dependent kinase inhibitor 2A; NRAS, Neuroblastoma RAS viral oncogene homolog; BRAF, B-Raf proto-oncogene, serine/threonine kinase; CRAF, C-Raf proto-oncogene, serine/threonine kinase; MEK, mitogen-activated extracellular signal-regulated kinase; ERK, Extracellular signal-regulated kinases; PI3K, Phosphoinositide 3-kinase; PIP3, Phosphatidylinositol (3,4,5)-trisphosphate; AKT, Ak strain transforming Protein kinase B; MDM2, Mouse double minute 2 homolog; PTEN, Phosphatase and tensin homolog; GF, Growth factor; ZEB, Zinc enter finger box-binding homeobox; NFkB, Nuclear factor kappa-light-chain-enhancer of activated B cells; BCL2, B-cell CLL/lymphoma 2 protein; EGFR, Epidermal growth factor receptor; IGF, Insulin-growth factor; TGFßR, Transforming Growth Factor beta receptor; FZD, Frizzled receptor; Wnt, Wingless-related integration site; MSH, melanocyte-stimulating hormone; MCR1-WT, melanocortin 1 receptor-wild type; cAMP, cyclic adenosine monophosphate; VEGFA, Vascular endothelial growth factor A; MMP, Matrix metalloproteinase; EMT, epithelial-mesenchymal transition; Rb, retinoplastoma protein; RbP, phosphorylated retinoblastoma protein; E2F, Early 2 Factor. *Created in BioRender. Hong, S. (2025)*

Dysregulated miRNAs play a crucial role in the development, progression, and treatment resistance of melanoma [151,152]. The central regulatory centers are the oncogenic microRNA (oncomiR) axis and the tumor suppressor network, and the critical downstream targets are the PTEN, MITF, MAPK, and angiogenesis pathways. Epigenetic silencing of tumor suppressor miRNA through DNA methylation and histone modifications is the drug target, leading to clinical trials combining demethylating agents with targeted therapies and immunotherapy. Fig. 2 illustrates the mechanisms of miRNA involvement in the signaling pathways of melanoma. The early and late stages of the disease, along with tumor heterogeneity, influence the paradoxical, positive, and negative regulatory feedback loops that contribute to the fluid and complex nature of melanoma pathogenesis.

OncomiRs/Tumor Suppressor miRNAs in Melanoma

Key oncomiRs consistently upregulated include miR-21 [153,154], miR-221/222 [155], miR-214 [156], miR-155 [157], and miR-18a [158] which promote proliferation, invasion, and drug resistance. In contrast, the tumor-suppressing miRNAs that were downregulated consist of miR-200c [159], miR-205 [160], miR-211 [161,162], miR-137 [163], miR-34a/b [164,165], the miR-29 family [166], and the let-7 family [167], which typically inhibit EMT, regulate the cell cycle, and restrain metastasis. miRNA-155 has both oncogenic and tumor suppressive roles.

Specific miRNAs in Melanoma Development

The major specific miRNAs that are crucial for melanoma development are 1) miRNA-21, a master oncomiR targeting multiple tumor suppressors [153,154] 2) miR-221/222 cluster, cell cycle and differentiation regulators [155] 3) miR-29 family, MAPK-responsive tumor suppressors [166] 4) miRNA-34a, p53-regulated master tumor suppressor [164] 5) miRNA-211, paradoxical behavior in melanoma [161,162]; 6) Let-7 family, tumor suppressors targeting RAS and cell cycle genes [167].

miRNA Targets in Signaling Pathways

**PTEN-PI3K/AKT axis:** Metastasis-associated microRNAs, miR-18a-5p, miR-93-5p, and miR-155-5p, target PTEN collectively, which activates the PI3K/AKT signaling pathway, facilitating melanoma invasion and metastasis [168]. OncomiRs such as miR-21 directly target PTEN, activating the AKT pathway and reducing regulatory T cell (Treg) proliferation [152]. Tumor-secreted miR-214 stimulates Tregs to produce IL-10 by lowering the levels of PTEN. This leads to immune suppression and accelerated tumor growth. Blocking miR-214 has been shown to inhibit Treg activity and slow tumor progression [169]. Up-regulated miR-146a in melanoma accelerates tumor cell growth by activating the NOTCH/PTEN/AKT pathway [170]. The loss of PTEN due to miRNA dysregulation also contributes to resistance to anti-PD-1 immunotherapy by maintaining PI3K/AKT pathway activation, suggesting that a combined approach targeting both PI3K and the miRNA-PTEN interaction may overcome therapeutic resistance in melanoma [171]. Interestingly, miR-92b-3p is the most abundant miRNA in melanoma-derived extracellular vesicles and directly inhibits PTEN, thereby promoting the formation of cancer-associated fibroblasts [172].

**MITF-miRNA regulatory network:** Tumor-suppressing miRNAs such as miR-148a/b [173], miR-137 [174], and miR-101-3p [175] inhibit melanoma progression by directly targeting MITF. Conversely, MITF influences specific miRNAs, such as miR-211 [161] and miR-579-3p [176], creating feedback mechanisms that govern the shift between proliferative and invasive traits. These MITF-miRNA circuits are increasingly recognized as mediators of BRAF/MEK inhibitor resistance, with the MITF-miR-579-3p axis emerging as both a mechanistic driver and predictive biomarker [176]. An interesting data-driven network approach revealed that specific miRNAs-31, 107, and 222-significantly influence melanoma metastasis and invasion, both individually and in combination, by modulating SOX10, MITF, and their shared targets through various direct and indirect interactions [177].

**MAPK pathway modulation:** Several miRNAs have been identified that either promote resistance (miR-514a, miR-1246, miR125b) or restore sensitivity to BRAF and MEK inhibitors (miR-7, miR-200c, miR-524-5p, miR-579-3p) by regulating genes involved in autophagy or the RAS/MEK/ERK pathway [178–180]. The oncosuppressor miR-579-3p and the MITF positive feedback regulatory loops govern the balance between proliferation, senescence, and therapeutic resistance in BRAF-mutant melanomas [176]. miR-579-3p and miR-1246 represent promising therapeutic targets and biomarkers in BRAF-mutant melanoma due to their intersection with the MAPK pathway and roles in drug resistance through processes of autophagy and immune checkpoint regulation [181].

**p53 pathway and MDM2:** The p53-MDM2-miRNA regulatory network is essential for melanoma progression and the development of therapy resistance. Several oncosuppressive miRNAs, such as miR-579-3p, miR-145, miR-23a, and miR-34a, either target MDM2 or act as p53 transcriptional targets, thereby influencing tumor suppressor functions [182,183]. Small molecules that hinder the interaction between p53 and MDM2 can reactivate p53 transcriptional activity and increase levels of tumor-suppressive miRNAs, such as miR-145 and miR-23a [183]. MDM2 inhibitors show clinical promise for restoring p53 function [184] with emerging data supporting synergy with immunotherapy [185].

**Wnt/β-catenin signaling:** miR-137 functions as a tumor suppressor in uveal melanoma by targeting EZH2, which leads to the suppression of Wnt/β-catenin signaling and EMT [163]. In contrast, miR-942-5p acts as an oncomiR by directly inhibiting Dickkopf-3 (DKK-3), a suppressor of the Wnt pathway [186]. This causes increased nuclear accumulation of β-catenin, thereby aiding in melanoma cell proliferation and invasion.

**Angiogenesis pathway:** The concept of “angiogenic switch” describes the transition in the tumor microenvironment from a dormant, avascular state to an active state characterized by an increase of pro-angiogenic factors and a decrease of anti-angiogenic factors [187]. Several miRNAs influence endothelial cell activity, vascular endothelial growth factor (VEGF) signaling, and the tumor microenvironment. miRNA dysregulation may facilitate cell invasion and migration *in vitro* and promote the formation of new vasculogenic structures by melanoma cells, so-called vasculogenic mimicry, presumed to be the result of differentiation plasticity inherent to more primitive, melanoma stem-like cells [188]. miR-378a-5p increases VEGF levels and enhances both *in vitro* and *in vivo* angiogenesis [189]. miR-155, derived from melanoma exosomes, promotes the secretion of VEGFA and FGF2, and proteolytic enzymes [148,190].

Epigenetic Mechanisms Silence Tumor Suppressor miRNAs

DNMT1 silencing of miR-211 [191] and combined DNA methylation/H3K27me3 repressing of miR-34a are emerging melanoma-specific targets. Clinically available agents—decitabine, azacitidine, and HDAC/EZH2 inhibitors—offer therapeutic opportunities to restore tumor suppressor networks [192].

miRNA and Membrane Pumps

miRNAs may modulate the expression of membrane pumps responsible for drug efflux directly. An example of this involves the observation that diminution of miR-340-5p levels is related to increased expression of the multi-drug resistance transporter, ATP-binding cassette, sub-family B member 5 (ABCB5), in melanoma cells under oxygen-deprived conditions [193]. Because ABCB5 is expressed by melanoma stem cells [194] and its function also drives pathways that enhance tumor virulence [195], this effect has pleiotropic implications that impact on both therapy resistance and intrinsic tumor aggressiveness.

### 1.4 Chromatin Remodeling in Melanoma

#### 1.4.1 SWI/SNF Complexes: Structure and Function

SWItch/Sucrose Non-Fermentable (SWI/SNF) complexes are ATP-dependent chromatin remodelers that play a significant role in gene expression by modifying nucleosome positioning. They are essential for processes such as transcription, DNA replication, and repair. These complexes contain a central catalytic subunit, which can either be SWIF/SNF-related, matrix-associated, actin-dependent regulator of chromatin, subfamily a (SMARCA)4/Brahma-related gene 1 (BRG1) or SMARCA2/Biologic Response Modifier (BRM), along with various subunits. The complexes include canonical BRG1/BRM-associated factor (cBAF), polybromo-associated BAF (PBAF), and noncanonical BAF (ncBAF) [196,197]. SWI/SNF complexes are vital for melanocyte development and the cellular response to ultraviolet (UV) radiation [198]. Mutations in the genes that encode SWI/SNF subunits are found in 34% of melanomas, with some acting as tumor suppressors and others promoting oncogenesis.

#### 1.4.2 SMARCA4 Heterogeneity and Therapeutic Sensitivity

The impairment of SMARCA4 function may enhance melanoma cell proliferation, invasiveness, and treatment responses. The SMARCA4 status in melanoma may be heterogeneous, with some tumors showing a loss of SMARCA4 function, while in other cases, high levels of SMARCA4 may promote cancer development. The array of transcription factors found in melanoma could determine whether SMARCA4 acts as a tumor suppressor or an oncogene by influencing its genomic location. The activity of SWI/SNF complexes can affect the sensitivity of melanoma cells to different therapies [199]. In a melanoma model, the somatic loss of function of SMARCA4, with the subunit Bptf, suppressed tumor development and disrupted intersecting gene expression programs crucial for tumor cell growth [200]. Research involving uveal melanoma cell lines has demonstrated a response to SMARCA4/SMARCA2 inhibition through small-molecule inhibitors targeting BRM/BRG1 ATPase activity [201].

#### 1.4.3 Other SWI/SNF Subunits in Melanoma

In melanoma cells, BRAF V600E-mediated apoptosis is dependent upon SMARCB1 [202]. SMARCD1 and SMARCD2 both engage with MITF and could play significant roles in the development of melanocytes and the progression of melanoma [203]. SMARCD3 is associated with reduced survival rates in patients with uveal melanoma [204]. Further, AT-rich interaction domain 1A (ARID1A) is the most mutated SWI/SNF gene in cancer [205]. Melanoma patients whose tumors exhibit elevated levels of ARID1A show a higher rate of clinical response to immune checkpoint inhibitors [206]. ARID1B frequently exhibits loss of chromosomal copies in mucosal melanomas [207]. Mutations in ARID2 are linked to exposure to ultraviolet radiation and are observed during the progression to melanoma *in situ* [208]. A lack of ARID2 enhances the effectiveness of immune checkpoint inhibitors in melanoma by increasing the production of chemokines and facilitating T-cell infiltration [209]. PHD finger protein 10 (PHF10), a component of the PBAF complex, has been found to be highly expressed in cutaneous melanoma and interacts with MYC to stimulate cell proliferation [210]. Elevated levels of BRD7 correlate with reduced survival rates in melanoma patients [211]. BRD9 plays a complex role in melanoma. BRD9 is overexpressed in melanoma and is found to be inhibited by a small molecule, TP-472 [211]. The Nucleosome Remodeling Factor (NURF) complex, with its subunit Bromodomain PHD finger transcription factor (BPTF), is essential for epigenetic modification associated with progression of melanoma. Melanomas show an overexpression of BPTF, which correlates with an unfavorable prognosis and resistance to BRAF inhibitors. Experimental silencing BPTF in melanoma cells resulted in a 65.5% reduction in the proliferative capacity and a 66.4% decrease in the metastatic potential [212]. The loss of Alpha-thalassemia/mental retardation, X-linked (ATRX) has been linked to the progression of melanoma, with decreased levels of ATRX mRNA noted in metastatic cases [213]. In mucosal melanoma, ATRX loss or reduced expression is associated with tumor progression and the alternative lengthening of telomeres (ALT) pathway, while ATRX alterations are less frequent in cutaneous melanoma. An analysis of 21 melanoma cases found that ATRX mutations were connected to loss of ATRX protein expression and ALT, suggesting that ATRX alterations may represent an initial event in conjunctival melanoma development [214]. Furthermore, both ATRX loss and TERT promoter mutations are present in premalignant conjunctival melanocytic lesions, with a majority of metastatic cases exhibiting one of these changes [215].

### 1.5 RNA Modification in Melanoma

#### 1.5.1 Overview of RNA Modifications

Various RNAs can undergo post-transcriptional modifications that influence cellular stability, localization, and function [216]. Transfer RNAs (tRNAs) exhibit a wide range of modifications [217], while ribosomal RNAs (rRNAs) and non-coding RNA also show a variety of post-transcriptional modifications [218]. Messenger RNAs (mRNAs), similarly, have different types of internal modifications [219]. In melanoma, specific RNA modifications have been linked to tumor development and progression, including N6-methyladenosine (m6A) and adenosine-to-inosine (A-to-I) editing. The upregulation of m6A modification regulatory proteins and A-to-I editing alter the sequences of proteins.

#### 1.5.2 m6A Modification Machinery: Writers, Erasers, Readers

Modification of m6A is a reversible and tightly regulated process involving three types of enzymes and binding proteins—writers, erasers, and readers—that collaboratively control the specific placement of m6A on RNA. This process governs RNA functions such as nuclear transcription, export, cytoplasmic stability, translation, and spatial regulation [220]. Writers, such as Methyltransferase-like 3 (METTL3), METTL4, and RBM15/15B, serve as methyltransferases and form complexes that install m6A marks in the nucleus. Erasers, including AlkB homolog 5 (ALKBH5) and Fat mass and obesity-associated (FTO), act as demethyltransferases, removing these methyl groups to fine-tune their distribution [221]. Reader proteins, namely YT521-B homology domain (YTHD)C1/2 in the nucleus and YTHDF1/2/3 in the cytoplasm, interpret these marks to influence RNA splicing, transport, stability, and translation. Through their coordinated activity, these writer, eraser, and reader enzymes govern various aspects of RNA metabolism [222].

#### 1.5.3 m6A Modifications and Melanoma Progression

Wang et al. performed single-cell and spatial RNA-seq in T cells, which revealed that elevated m6A modification contributes to melanoma virulence and is associated with decreased immune cell infiltration; the expression of m6A readers (YTHDF2, RBM15B) and writers (METTL3, ZC3H13) was reduced, while the m6A eraser (FTO) was upregulated [223].

A study examining the epitranscriptomic profile of melanoma through several publicly accessible databases highlighted DNMT3A and METTL4 as key potential regulators of melanoma growth. Functional validation of DNMT3A and METTL4 was performed using shRNA-mediated knockdown, demonstrating that their depletion in melanoma cells resulted in inhibited cell growth [224].

A study showed that METTL3-mediated m6A hypermethylation of uridine-cytidine kinase 2 (UCK2) enhanced melanoma cell metastasis by activating the Wingless-related integration site (WNT)/β-catenin signaling pathway [225]; UCK2 is the key rate-limiting enzyme in the pyrimidine nucleotide salvage pathway, thus playing a significant role in promoting cancer cell invasion, proliferation, and metastasis [226]. Additionally, METTL3 promotes EMT and thereby promotes cancer cell migration and invasion [227]. Melanoma cells in particular exhibit elevated METTL3 expression compared to normal melanocytes, indicating that METTL3 may play a role in regulating proliferation, invasion, migration, and drug resistance of melanoma cells [228].

The mutation and expression levels of the ALKBH5 gene in melanoma patients are associated with their response to immunotherapy. Furthermore, a small-molecule inhibitor of ALKBH5 has been shown to improve the effectiveness of cancer immunotherapy [229]. ALKBH5 enhances the stability and expression of FOXM1 mRNA by removing m6A methylation, thereby inducing epithelial-mesenchymal transition (EMT) and promoting melanoma metastasis [230].

Research has shown that when FTO demethylates mRNA, it can stimulate the growth of melanoma and diminish the effectiveness of anti-PD-1 immunotherapy. When FTO was knocked down in mice, melanoma cells exhibited increased sensitivity to interferon-gamma and showed improved responses to anti-PD-1 therapy [231].

#### 1.5.4 A-to-I RNA Editing in Melanoma

A-to-I editing has been implicated in both the promotion and inhibition of tumor growth in melanoma. The editing of miR-455-5p by Adenosine Deaminase Acting on RNA 1 (ADAR1) may enhance metastasis, while editing of miR-378a-3p can suppress metastasis of melanoma. Alterations in A-to-I editing levels in melanoma cells have also been associated with resistance to apoptosis. Additionally, A-to-I editing can aid in immune evasion by altering the immunogenic properties of specific RNA structures, which hinders recognition by the immune system [232]. Furthermore, RNA editing signatures have been found within genes that can differentiate patients’ responses to immunotherapy among various patient groups [233].

#### 1.5.5 Future Directions Utilizing RNA Modification

Preclinical studies have highlighted the significant roles of both m6A and adenosine-to-inosine (A-to-I) RNA editing in various diseases, including cancer, chronic inflammation, and infections, fueling interest in their therapeutic applications. Structural insights into ADARs and key m6A enzymes have enabled the development of small-molecule inhibitors that modulate their expression, catalytic activity, or RNA binding. Additionally, targeting RNA modifications and their regulators may enhance the effectiveness of immunotherapies by modulating the tumor immune microenvironment [234].

## 2 Epigenetic Biomarkers in Diagnosis and Prognosis of Melanoma

### 2.1 Methylation-Based Biomarkers

#### 2.1.1 MGMT Promoter Methylation as Biomarkers

The most extensively studied epigenetic modifications in melanoma involve DNA methylation patterns, with multiple markers demonstrating diagnostic and prognostic value across independent cohorts [69]. Among all epigenetic markers, only MGMT promoter methylation currently plays a role in guiding therapeutic decisions for melanoma, although its adoption varies substantially among institutions [57]. Routine MGMT testing in melanoma centers remains limited, primarily because immunotherapy and targeted therapy have largely replaced chemotherapy as first-line treatments. Epigenetic silencing of MGMT may sensitize melanoma cells to alkylating agents such as temozolomide by impairing their ability to repair chemotherapy-induced DNA damage [235]. One study of patients with stage IV cutaneous melanoma showed that MGMT promoter methylation is associated with a significantly improved response rate to the single-agent dacarbazine/temozolomide [80]. The DeCOG study found no significant association between MGMT promoter methylation status and treatment response, progression-free survival, or overall survival in melanoma [236]. MGMT promoter methylation may help identify melanoma patients who would benefit from melphalan regional chemotherapy [237].

#### 2.1.2 RASSF1A Hypermethylation as Biomarkers

RASSF1A hypermethylation serves as a diagnostic and prognostic marker in melanoma. Melanoma cell lines show 69% methylation [238], and patient tumors demonstrate a striking stage-dependent pattern, occurring in 0% of stage I/II primary melanomas increasing to 26.7%–27.8% in stage III disease and 48.9% in stage IV metastatic melanoma [71,72,239]. RASSF1A methylation exhibits limited independent prognostic significance, as evidenced by TCGA analysis of 355 patients, which revealed no significant association with disease-free survival (HR = 0.94, *p* = 0.694) or overall survival (HR = 0.74, *p* = 0.106). However, it may predict a poor biochemotherapy response when highly methylated [240]. Methylation of the RASSF 1 A promoter in cell-free DNA is detected more frequently in melanoma patients than in healthy controls. Evaluation of this marker in plasma revealed high diagnostic accuracy for melanoma, with an area under the curve of 0.905. Although there is no significant correlation between cell-free DNA methylation and circulating tumor cells individually, combining both biomarkers improves the detection rate for invasive and metastatic melanoma [241].

#### 2.1.3 CDKN2A Promoter Methylation as Biomarkers

Approximately 25% of cutaneous melanoma metastases show hypermethylation of the CDKN2A promoter, with p16(INK4A) promoter methylation specifically detected in 15 of 59 metastatic cases. This alteration is significantly more common in NRAS-mutated tumors than in those without NRAS mutations (*p* = 0.0004), suggesting a link between NRAS mutations and p16(INK4A) methylation [242]. Methylation-induced loss of p16 function is an independent predictor of poor survival (HR 2.5) [243] and is closely associated with more aggressive clinical features, NRAS mutations, and the transition from primary to metastatic melanoma.

#### 2.1.4 CpG Island Methylator Phenotype (CIMP) as Biomarkers

**Figure 3:**
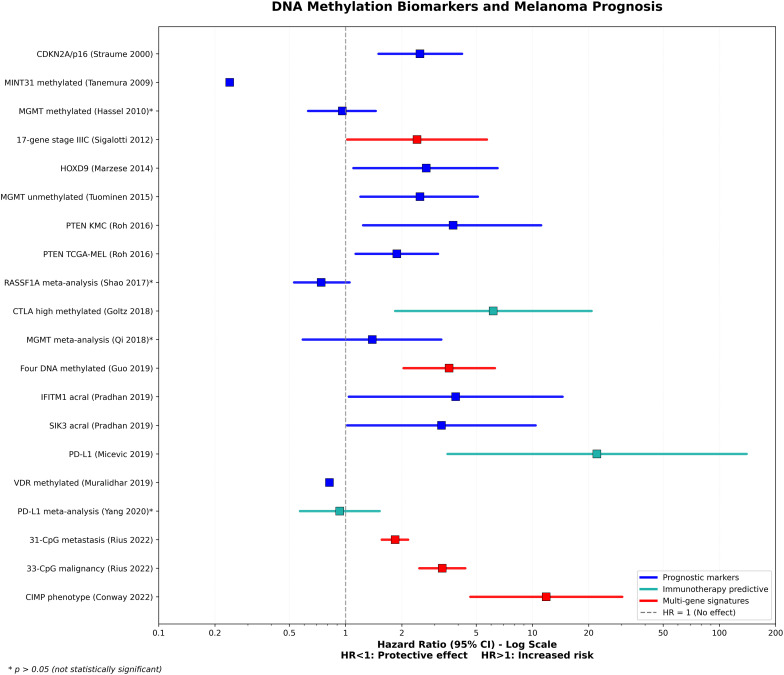
DNA methylation biomarkers and melanoma prognosis. Most DNA methylation biomarkers demonstrate increased mortality risk (Hazard Ratio [HR] > 1), with immunotherapy-predictive markers showing particularly strong associations—Programmed death-ligand 1 (PD-L1) [245] expression (HR = 22.1) and Cytotoxic TLymphocyte-Associated Protein 4 (CTLA-4) [246] high methylation (HR = 6.16). A multi-gene signature, CpG Island Methylator Phenotype (CIMP) [244] phenotype, indicates an elevated risk (HR = 11.84). Among individual prognostic markers, Phosphatase and TENsin homolog (PTEN) [247] and homeobox D9 (HOXD9) [248] show moderate risk associations, while Methylated-IN-Tumour (MINT)31 [239] and Vitamin D Receptor (VDR) [249] methylation demonstrate protective effects (HR < 1) [80,236,240,243,250–255]. CI: Confidence Interval

The CIMP identifies melanoma patients with an increased risk of death, with a hazard ratio of 11.84 (95% CI: 4.65–30.20) compared to tumors with low methylation. CIMP-positive melanomas are more frequently observed in patients aged 65 or older, are associated with lentigo maligna melanoma histology, ulceration, advanced AJCC stage, and a lower number of tumor-infiltrating lymphocytes [244]. The CIMP gene panel consists of Methylated-IN-Tumour (MINT)17, MINT31, Tissue factor pathway inhibitor 2 (TFPI2), Wnt inhibitory factor 1 (WIF1), RASSF1A, and Suppressor of cytokine signaling 1 (SOCS1) [239] as well as further hypermethylation observed in PTEN, Vitamin D Receptor (VDR), PD-L1, and TET2 [244]. Importantly, CIMP may be detectable as early as stage 1b, indicating its potential value for early risk assessment, if confirmed in future studies. Fig. 3 illustrates a forest plot of the meta-analysis of various DNA methylation biomarkers that are relevant to melanoma prognosis. Most of these biomarkers indicate an increased risk of mortality (Hazard Ratio [HR] > 1).

#### 2.1.5 5-hmC as a Diagnostic Biomarker

Identifying dependable ancillary markers to assist in the classification of melanocytic lesions continues to be a significant challenge. One of the most promising and well-documented epigenetic biomarker candidates is 5-hmC, an oxidized form of 5-methylcytosine produced by the TET family of dioxygenases. In benign melanocytic nevi, nuclear 5-hmC is expressed in high levels, but there is a progressive decrease in dysplastic nevi, Spitz tumors, atypical borderline melanocytic lesions, and invasive melanomas [44,58,256,257]. This gradual loss has been documented in various independent studies, suggesting that 5-hmC serves as an epigenetic marker for malignant transformation in melanocytes.

Extensive retrospective research has validated the diagnostic utility of 5-hmC immunohistochemistry. Rodic et al. found that employing a semi-quantitative 5-hmC scoring system could differentiate between nevi and melanomas, achieving over 90% sensitivity and specificity [256]. Further investigations by Yu and colleagues corroborated these results, indicating that 5-hmC and PRAME exhibit complementary expression patterns, with the loss of 5-hmC combined with diffuse PRAME positivity being particularly indicative of melanoma [258]. In their study of 144 lesions, the area under the ROC curve (AUC) was 0.91 for 5-hmC alone and improved to 0.97 when paired with PRAME, emphasizing the advantage of using these markers together.

Despite its diagnostic value, interpreting 5-hmC results does come with challenges. 5-hmC is a fundamental epigenetic regulatory modification that is ubiquitously present across cell types and is not lineage-specific. This often necessitates double labeling by immunohistochemistry (IHC) with melanocytic markers, such as Melanoma Antigen Recognized by T cells (MART)-1, to accurately assess 5-hmC level in the melanocytic lesional cells [259,260]. Moreover, 5-hmC staining typically shows a continuous gradient rather than a clear dichotomy, compromising the reproducibility of semi-quantitative scoring across different observers [47,261]. Additional internal control issues may surface due to 5-hmC regulation in keratinocytes and neighboring epithelial cells, with adjacent squamous dysplasia or actinic damage potentially complicating interpretations [262,263]. These complications might explain the variable levels of 5-hmC in melanocytic neoplasms. In addition, the understanding of mechanisms of loss 5-hmC in melanoma remains incomplete. We observed that specific melanoma subtypes, such as acral or desmoplastic melanoma, do not exhibit significant loss of 5-hmC compared to other melanoma subtypes, and the underlying mechanism remains unclear [264,265].

To address subjective biases, recent studies have utilized computer-assisted digital image analysis. Research of standardized image quantification in dysplastic nevi and superficial spreading melanomas revealed that 5-hmC levels in the epidermal/junctional area offered the most significant differentiation, with ROC-AUC values between 0.76 and 0.79 [266]. This work highlights the potential of digital pathology, supported by AI-assisted algorithms, to enhance reproducibility and enable more precise reporting frameworks. Similar methodological improvements have been investigated in other groups [267,268].

**Figure 4:**
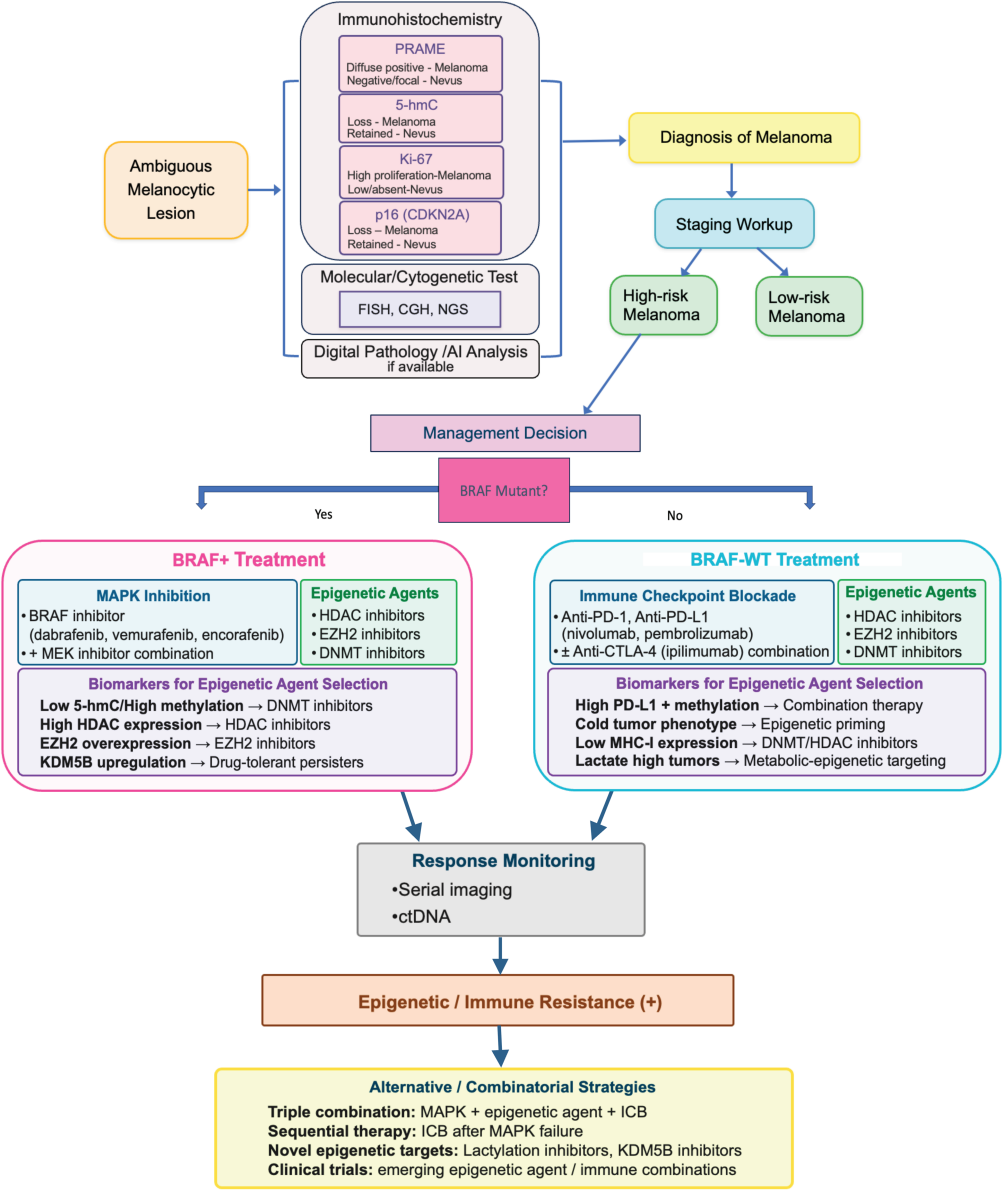
Integration of epigenetics into diagnosis and management of melanoma. An ambiguous melanocytic lesion diagnosis may benefit from epigenetic biomarkers as ancillary tests. Management of high-risk melanoma may include epigenetic agents with clinical trial enrollment, and epigenetic biomarkers for treatment selection. Abb: 5-hmC, 5-hydroxymethylcytosine; PRAME, Preferentially Expressed Antigen in Melanoma; CDKN, Cyclin-Dependent Kinase Inhibitor; FISH, Fluorescence *In-Situ* Hybridization; CGH, Comparative Genomic Hybridization; NGS, Next-Generation Sequencing; AI, Artificial Intelligence; ctDNA, Circulating tumor DNA; HDAC, Histone Deacetylase; DNMT, DNA Methyltransferase; EZH2, Enhancer of Zeste Homolog2; KDM5B, Lysine Demethylase 5B; MHC, Major Histocompatibility Complex; MAPK, mitogen-activated protein kinase; BRAF-WT, B-Raf proto-oncogene, serine/threonine kinase-wild type; MEK, mitogen-activated extracellular signal-regulated kinase; PD-1, programmed cell death protein-1; PD-L1, programmed death-ligand1; CTLA-4, Cytotoxic T-lymphocyte-associated protein-4; ICB, Immune Checkpoint Blockade

Overall, the evidence strongly endorses 5-hmC as a beneficial addition to the histopathological assessment of melanocytic tumors. Its optimal diagnostic utility lies best in conjunction with other markers such as PRAME [258], p16 [58], and Ki-67 [264]. Fig. 4 illustrates a workflow diagram of the integration of epigenetics into the diagnosis of an ambiguous melanocytic lesion and treatment of high-risk melanoma.

Moreover, 5-hmC has prognostic and mechanistic relevance [263]: its reduction is associated with increased tumor aggressiveness and adverse outcomes, and reinstating 5-hmC via TET/IDH pathways reduces melanoma growth in experimental settings [44,269]. As digital pathology and molecular testing advance, incorporating 5-hmC into multi-parameter panels shows great promise for enhancing diagnostic precision [263], aiding in risk assessment, and potentially guiding therapeutic innovations in melanoma.

### 2.2 MicroRNA Panels for Early Detection/Relapse Prediction

Recent advancements in miRNA-based liquid biopsy techniques have yielded encouraging results for the early detection and relapse prediction in melanoma [145].

#### 2.2.1 Diagnostic miRNA Panels

Circulating miRNA biomarkers have demonstrated a sensitivity of 87% and specificity of 81% in various meta-analyses [270]. Several validated panels have been developed, including the MEL38 signature, which consists of 38 circulating plasma miRNAs and has shown strong independent classification accuracy (AUC 0.79–0.94) when analyzed using a support vector machine algorithm [271]. The MEL38 diagnostic signature classifies patients into distinct diagnostic categories using RNA sequencing from either solid tissue or plasma. MEL38 scores were able to differentiate between metastatic and locally invasive melanoma samples, as well as between melanoma *in situ* and normal skin samples. However, MEL38 scores could not differentiate between benign nevi and melanoma *in situ* samples [272].

A specific 4-miRNA plasma extracellular vesicle panel has shown to non-invasively diagnose melanoma with an AUC ranging from 0.75 to 1.00 [273]. A meta-analysis and systematic review conducted by Jones and Nonaka evaluated 898 melanoma patients across 9 studies using circulating miRNA panels and reported 89% sensitivity, 85% specificity, a diagnostic odds ratio of 45, and an AUC of 0.93 [274].

#### 2.2.2 Prognostic miRNA Panels

Friedman et al. demonstrated that serum miRNAs may be used as biomarkers for both monitoring melanoma tumor burden over time and accurately identifying primary melanoma patients at elevated risk of recurrence [275]. A study by Stark et al. highlighted that the prognostic and predictive capabilities of the MELmiR-7 panel have been shown to predict the overall survival of melanoma patients more effectively than both serum lactate dehydrogenase (LDH) and S100B levels. microRNAs associated with melanoma [276]. Moreover, a combination of four miRNAs (miR-150, miR-30d, miR-15b, and miR-425) was effective in differentiating patients based on recurrence-free and overall survival, improving the prediction of recurrence beyond stage classification. It was observed that serum miR-15b levels increased significantly over time in patients who experienced recurrence, while in non-recurrent patients, these levels did not show significant changes over time. The demonstration that miR-15b rises progressively only in recurrence cases provides biological evidence for a window of opportunity where enhanced surveillance or earlier therapeutic intervention may improve outcomes [277]. In the case of solid tissue, the prognostic MEL12 signature categorizes patients into low-, intermediate-, and high-risk groups, regardless of clinical covariates. The hazard ratios for 10-year overall survival, based on survival intervals, were found to be 2.2 (high-risk vs. low-risk) and 1.8 (intermediate-risk vs. low-risk), surpassing those of other existing prognostic models [272]. Lastly, a study identified specific circulating cell-free miRNAs in plasma samples from melanoma patients with brain metastasis, which may provide insights into assessing those at risk for developing melanoma brain metastasis [278].

### 2.3 Liquid Biopsy and Circulating Epigenetic Markers

#### 2.3.1 Overview and Current Landscape

The liquid biopsy field is undergoing rapid advancements, with 2024 marking a high point in published research on the subject [279]. Blood remains the primary biofluid studied, with a focus mainly on circulating tumor DNA (ctDNA) as the most extensively analyzed component. Other significant components include circulating tumor cells (CTCs), nucleosomes, lncRNAs, and miRNAs, which are found in exosomal vesicles or as cell-free RNA [280].

#### 2.3.2 ctDNA vs. miRNA: Comparative Advantages

Unlike ctDNA, which is released from necrotic or apoptotic tumor cells and consequently may lead to low and inconsistent levels in circulation, circulating miRNA can be released by cancer cells regardless of their viability. ctDNA has a short half-life of less than two hours, which can complicate laboratory processes such as sample preparation, storage, and transportation, potentially affecting the accuracy of the results. In contrast, miRNAs are stable in circulation since they are packed in exosomes or encapsulated lipid particles and may also be protected by nucleophosmin, microvesicles, and high-density lipoproteins [145].

#### 2.3.3 Advantages and Limitations of Epigenetic Liquid Biopsy

The advantages of utilizing epigenetic modifications in liquid biopsies include their ability to provide early insights into tumor development, details about tissue origin, diverse features, and the potential for reversibility and dynamism. The disadvantages include limitations due to low quantities and a lack of standardized detection and analysis methods [281]. Research on epigenetic liquid biopsy in melanoma is not as extensive as in other cancers, such as breast, colorectal, lung, and ovarian cancers. Other cancer research has yielded promising findings regarding ctDNA of 5-mC and 5-hmC, as well as nucleosome and histone post-translational modifications, ncRNAs, and miRNAs. Multiple epigenetic markers are often combined for clinical application [281].

#### 2.3.4 ctDNA as a Prognostic Marker in Melanoma

The prospective PET/LIT study enrolled 104 patients and demonstrated that ctDNA has a prognostic value in patients with advanced melanoma treated with immune checkpoint inhibitors. Using patient-specific mutations, tumor-informed panels are designed in 1 week, and ctDNA can be sequenced and analyzed in 2 weeks, enabling personalized monitoring. Although the cohort size was small, ctDNA was detected in 76.9% of adjuvant patients who experienced relapse, whereas all patients without disease progression remained ctDNA-negative [282]. In a prospective clinical trial of ctDNA for advanced melanoma, post-surgery positive ctDNA was associated with worse overall survival (HR 6.04) and systemic relapse-free survival (HR 3.77), with ctDNA detection preceding radiological relapse by a median of 4 months [283].

#### 2.3.5 Emerging Biosensor Technologies

New advances in liquid biopsy introduce biosensor technologies enabling point-of-care testing with nanomolar detection limits. Optical biosensors offer a significant opportunity in liquid biopsy due to their enhanced sensing performance and practical, user-friendly properties. The technology has demonstrated sensitive detection of proteins, peptides, ctDNA, miRNA, exosomes, and CTCs [284]. The distinct mutations of ctDNA make them detectable targets via electrochemical methods, and small non-coding RNAs have been the focus of electrochemical detection due to their differential expression in cancer cells [285].

## 3 Epigenetic Therapy in Melanoma

### 3.1 Epigenetic Therapeutic Agents

**Figure 5:**
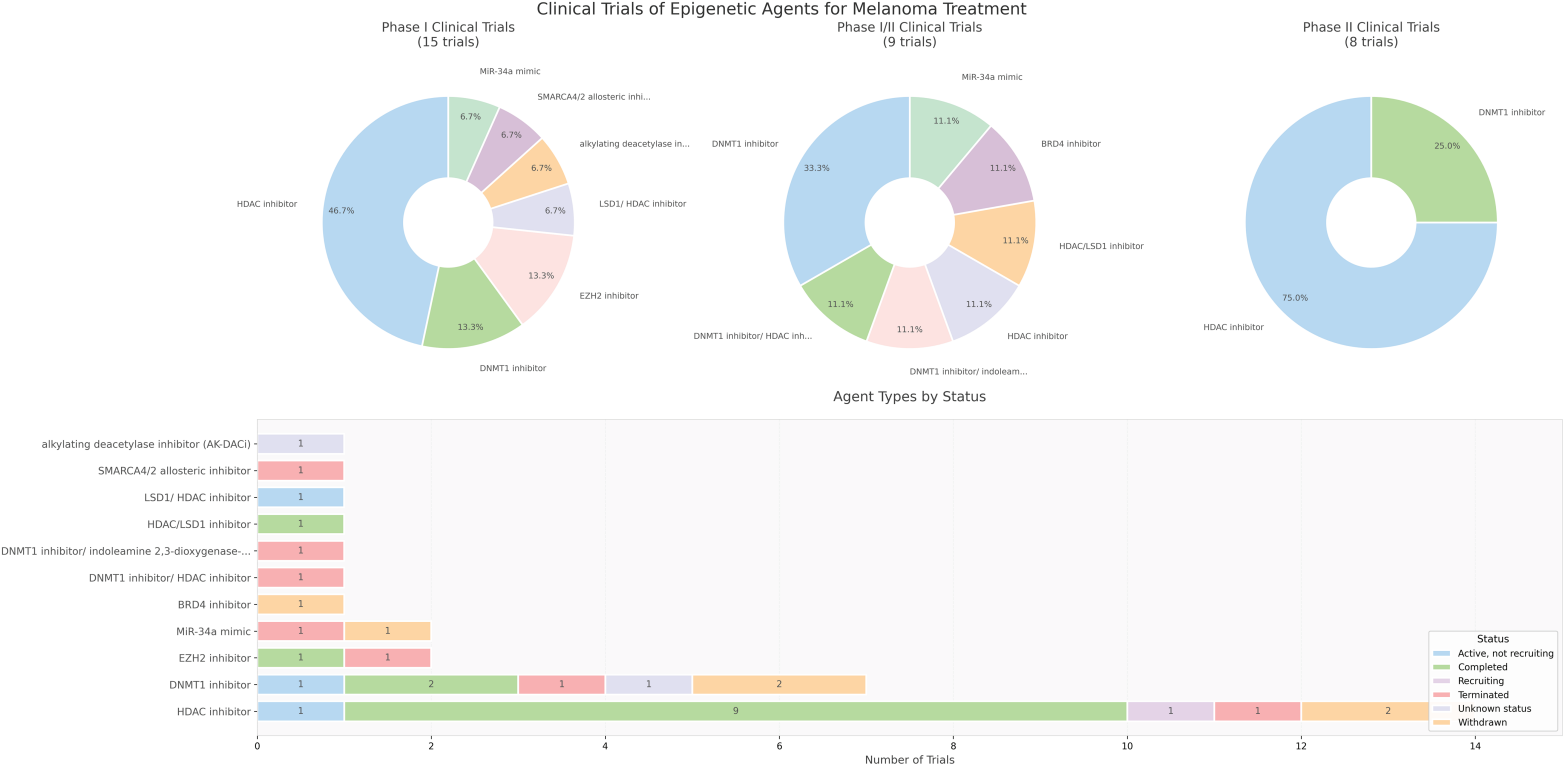
Distribution of current clinical trials of epigenetic agents for melanoma treatment. Phase graphs show the distribution of agents in each phase, and the bar graph shows the trial status of each epigenetic agent

The effectiveness of epigenetic drugs as single agents in solid tumors remains limited, unlike their significant clinical efficacy in certain hematological malignancies [286]. The clinical trials have revealed an unexpectedly high incidence of side effects that need further scrutiny. The major epigenetic drugs that are in clinical trials are DNMT inhibitors, HDAC inhibitors, and EZH2 inhibitors (Fig. 4). Table A2 lists the recent clinical trials of epigenetic agents for melanoma. HDAC inhibitors are the majority of all the phase I, I/II, and II trials, followed by DNMT1 inhibitors (Fig. 5). Currently, there are no epigenetic drugs that have received FDA approval specifically for the treatment of melanoma.

DNMT inhibitors are designed to inhibit the activity of DNA methyltransferases, leading to the reactivation of silenced tumor suppressor genes. Preclinical studies have shown that these inhibitors can re-sensitize melanoma cells to conventional chemotherapy and targeted therapies. However, clinical trials of these agents in melanoma have shown mixed results, highlighting the need for more specific and effective agents [192]. HDAC inhibitors (HDACi) are among the most well-explored epigenetic drugs for melanoma treatment. These inhibitors can induce histone hyperacetylation, leading to chromatin relaxation and reactivation of tumor suppressor genes.

HMT inhibitors, such as EZH2 inhibitors, are emerging as a potential therapeutic strategy. By inhibiting EZH2, these drugs aim to reactivate the expression of tumor suppressor genes, thereby slowing melanoma progression and metastasis. Early phase clinical trials are investigating the safety and efficacy of these inhibitors in combination with other treatments [192]. JQ1, an inhibitor of BRD4, shows promise in enhancing immunotherapy for melanoma by modulating immune responses and inhibiting tumor progression. It can suppress the expression of c-MYC and PD-L1, which may lessen tumor-induced immunosuppression and enhance the effectiveness of treatments such as PD-1/PD-L1 blockade [287]. JQ1 has also been investigated to enhance the apoptosis of B16 melanoma cells by modifying mitochondrial dynamics, which leads to mitochondrial dysfunction and an elevation in oxidative stress [288]. Targeting non-coding RNAs is another exciting avenue for epigenetic therapy in melanoma. miRNA-based therapeutics are being developed to either mimic tumor-suppressing miRNAs or inhibit oncogenic miRNAs [289].

### 3.2 Combination Strategies with Immunotherapy and Targeted Therapy

#### 3.2.1 Overview of Epigenetic Combination Approaches

Epigenetic agents have been combined with chemotherapies, immunotherapies, and targeted therapies, including other epigenetic drugs, to target parallel tumorigenic pathways, enhance cancer cell death, and overcome resistance mechanisms. Despite these efforts, epigenetic combination therapies have achieved limited success in solid tumors. The underlying cause of poor efficacy may suggest that solid tumors are biologically more complex or are less reliant on epigenetic modifications [286].

Clinical trials investigating HDACi in combination with other melanoma therapies are showing some therapeutic efficacy [192]. While Vorinostat has not received FDA approval for melanoma, patients with BRAFi/MEKi-resistant BRAFV600-mutated melanoma have tolerated intermittent treatment with vorinostat well, and 9% of these patients experienced lasting antitumor responses [290].

#### 3.2.2 Emerging Epigenetic Targets for Combination Strategies

Novel epigenetic treatment such as targeting PRMT1 may be considered for synergistic combination therapy with checkpoint inhibitors, as PRMT1 has shown to enhance interferon signaling by increasing the levels of endogenous retroviral elements [291]. Another new insight demonstrates how understanding epigenetic resistance mechanisms can lead to effective, low-toxicity therapeutic strategies using FDA-approved drugs, such as statins, and suggests that Peroxisome proliferator-activated receptor-gamma coactivator 1-alpha (PGC1α alpha) [292] status could be routinely included in molecular profiling to guide treatment decisions [293].

#### 3.2.3 Clinical Trials for Checkpoint Inhibitor-Refractory Patients

For patients with cutaneous melanoma who have progressed or are refractory to checkpoint inhibitor therapy, class I HDAC inhibitor (entinostat or domatinostat) + anti-PD-1 rechallenge has been tried. The SENSITIZE trial with Domatinostat + Pembrolizumab objective response rate (ORR) is 7.5% and disease control rate (DCR) is 30% (NCT03278665. Table A2). The ENCORE-601 trial with Entinostat + Pembrolizumab did not achieve the primary response rate endpoint but provided a clinically meaningful benefit, with objective response in 9% of patients. No new toxicities, including immune-related adverse events, were seen for either drug [294]. The rationale centers on the ability of HDAC inhibitors to enhance tumor antigen presentation and reverse immune evasion mechanisms.

#### 3.2.4 Strategies for BRAF/MEK Inhibitor-Resistant Melanoma

For patients with BRAF-mutant melanoma who have developed resistance to BRAF/MEK inhibitor, HDAC inhibitor or DNMTi + continued/rechallenge of BRAF/MEK inhibitors has been tried. The most recent trial regimen is Tazemetostat + BRAF/MEK inhibitors [NCT04557956. Table A2]. For carefully selected treatment-naive patients with high-risk features, investigational approaches have been considered. For low tumor mutational burden (TMB) with hypermethylation signature, DNMTi + immunotherapy has been considered. For “Cold” tumor phenotype, HDACi + immunotherapy has been considered.

#### 3.2.5 Current Status of Epigenetic Agents

Until definitive Phase III validation becomes available, epigenetic agents remain as investigational tools that expand treatment options for patients with limited alternatives.

The integration of epigenetic therapies into melanoma treatment represents an evolving frontier that requires continued research, careful patient selection, and thoughtful clinical implementation within appropriate investigational frameworks.

### 3.3 Clinical Trial Failure Analysis

#### 3.3.1 Reasons for Clinical Trial Failures

Epigenetic drugs have not received FDA approval for melanoma treatment, despite clear pharmacodynamic activity. The main reasons for failure were the lack of patient selection biomarkers, use of maximum tolerated doses that caused excessive toxicity before reaching optimal biological doses, and the misconception that monotherapy would be effective in a disease that requires combination therapy. Single-agent trials showed low objective response rates. However, when these agents were combined with checkpoint inhibitors, response rates improved and remissions were more durable. These results indicate that strategic combinations guided by predictive biomarkers are a more promising approach than abandoning epigenetic therapies.

#### 3.3.2 Mechanisms of Immune Evasion in Cancer

Recognizing how cancer evades the immune system and discovering the concept of immune priming marked a critical turning point in the development of new treatment strategies. Immune evasion by cancer cell is explained by various mechanisms. Tumors promote an embryonic-like gene expression pattern in endothelial cells through angiogenesis, thereby hindering leukocyte infiltration and weakening antitumor immunity [295]. Immune evasion also starts from abnormal tumor blood vessels reducing blood flow and intensifying existing hypoxia within the tumor [296]. This hypoxic environment not only increases resistance to chemotherapy and radiation but also promotes immune escape by amplifying the activity of regulatory T cells, myeloid-derived suppressor cells, and tumor-associated macrophages [297].

#### 3.3.3 Epigenetic Immune Priming Strategy

Preclinical and early clinical studies have shown that several epigenetic therapies can prime the immune system, both as monotherapies and in combination with immune-based treatments [298]. Furthermore, metronomic chemotherapy has been shown to reduce immunosuppressive cells in the tumor microenvironment, such as regulatory T cells and myeloid-derived suppressor cells. Immune checkpoint inhibitors complement this effect by sustaining T cell activation and boosting anti-tumor responses. When combined, these strategies work synergistically to strengthen anti-tumor immunity [299]. Importantly, low-dose epigenetic priming with DNMTi and HDACi has proven more effective than standard maximum tolerated or prolonged dosing in melanoma, achieving sustained immunomodulation without cytotoxicity [300]. This was demonstrated in the NIBIT-M4 trial, where guadecitabine (30–60 mg/m^2^ SC, days 1–5) combined with ipilimumab resulted in a five-year overall survival rate of 28.9% [301].

#### 3.3.4 Future Directions of Trial Design

Appropriate patient selection using validated predictive biomarkers (methylation signatures, immune phenotypes, genetic markers), optimal biological dose-finding replacing maximum tolerated dose, pharmacodynamic monitoring ensuring target engagement, and rational combinations addressing complementary resistance mechanisms can transform these agents from categorical failures into precision medicine tools for melanoma populations. Fig. 4 illustrates an algorithm of how epigenetic biomarkers are used for treatment selection, and how epigenetics agents are used as combination regimens.

#### 3.3.5 Theoretical Biomarker-Driven Trial Schemas

Incorporating these corrective ideas, some theoretical biomarker-driven trial schemas may be: 1) Patients with high-methylation cluster tumors with IDH1 mutations may be treated with decitabine, followed by pembrolizumab. Serial ctDNA methylation monitoring can measure treatment effect, with non-responders crossing over to alternative therapy. 2) Patients with high-methylation at immune gene promoters with low PD-1 expression tumor may be treated with guadecitabine priming combined with ipilimumab then maintenance nivolumab. Serial 5-hmC increase can monitor treatment effect. 3) Patients with high HDAC2 expression with histone acetylation capacity assessment may be treated with panobinostat combined with pembrolizumab for checkpoint inhibitor rechallenge.

## 4 Artificial Intelligence (AI)/Integrative Multiomics Approach

AI and machine learning (ML) are revolutionizing melanoma management, particularly in biomarker discovery, patient stratification, and outcome prediction. This transformation is achieved through the integration of multi-omics data with sophisticated algorithmic techniques.

### 4.1 AI/ML for Biomarker Discovery

One notable approach is a transfer learning-based biomarker discovery model combined with an ensemble machine learning model. This innovative strategy successfully identified novel biomarkers, yielding an impressive AUC of 0.9861 and an overall accuracy of 91.05%. The study highlighted specific genes essential for diagnostic classification and identified both diagnostic and prognostic biomarkers specifically for melanoma [302].

### 4.2 AI/ML for Prognostic Stratification

In another significant advancement, researchers developed a machine learning-driven signature (MLDS) based on marker genes from various molecular subtypes. This signature notably enhanced predictive accuracy for melanoma patient prognosis. Findings indicated that MLDS scores were associated with decreased immune cell infiltration and lower expression of immune checkpoints. Patients categorized in the low MLDS group demonstrated greater responsiveness to chemotherapy and could potentially benefit more from immune checkpoint inhibitors. This study emphasizes the complexity of melanoma’s molecular subtypes and the critical role of the tumor microenvironment in disease progression [303].

### 4.3 ML with Multi-Omics Approach

Korfiati’s analysis utilized ML to integrate miRNA expression profiles with multi-omics, clinical, and imaging data, leading to a deeper understanding of biological processes and the identification of more informative biomarkers. The study demonstrated that miRNA signatures could be employed in training classification models to predict melanoma recurrence and metastasis with high accuracy rates of 91.51% and 97.39%, respectively. Notably, when clinical information was added, the predictive accuracy for melanoma recurrence in an external test group increased from 73.85% to 85.38% [304].

Another study employed weighted gene co-expression network analysis (WGCNA) combined with consensus clustering and 10 ML algorithms to develop an immunotherapy-related gene model (ITRGM). This multi-omics analysis, which included both bulk and single-cell RNA sequencing of melanoma patients, identified 66 consensus immunotherapy prognostic genes (CITPGs). The CITPG-high group not only exhibited improved prognosis but also showed enhanced immune activity. By utilizing a combination of ML algorithms, the ITRGM signature effectively stratified patients into high-risk or low-risk categories for immunotherapy response, serving as a reliable predictor for classifying melanoma patients into ‘immune-hot’ and ‘immune-cold’ tumors and thus enhancing immunotherapy outcomes [305].

A study focused on assessing machine learning algorithms for predicting melanoma recurrence using clinical and histopathologic data obtained from Electronic Health Records (EHRs). The study achieved recurrence classification performance with AUC values of 0.845 and 0.812, highlighting Breslow tumor thickness and mitotic rate as the most predictive features. These findings indicate that machine learning can effectively extract valuable predictive signals from clinicopathologic data, potentially leading to the identification of early-stage melanoma patients who may benefit from adjuvant immunotherapy [306].

Lastly, a recent study demonstrates that deep learning analysis of histopathology can serve as a spatiotemporal framework for optimizing the design and timing of epigenetic interventions. This approach enables the targeted elimination of resistant cancer clones, potentially improving treatment outcomes [307].

## 5 Challenges and Future Perspectives

Despite vast publications on melanoma epigenetics, current treatment guidelines rely solely on genetic mutations (BRAF/NRAS), tumor mutational burden, and PD-L1 expression. As of 2025, no epigenetic biomarkers have been included in standard clinical recommendations for melanoma. This significant gap between robust research and clinical use results from distinct, solvable barriers.

The main obstacle is the lack of prospective validation. Most studies on epigenetic biomarkers employ retrospective designs and small sample sizes, resulting in preliminary findings. Large, Phase III prospective trials, ideally multi-center and enrolling more than 500 patients, are needed to demonstrate that using epigenetic markers to guide treatment improves outcomes compared to standard care. While ongoing trials combining EZH2, HDAC, or DNMT inhibitors with immunotherapy or targeted therapy may eventually provide this evidence, results are still pending.

Technical standardization issues make reproducibility between labs difficult. Assay platforms, including methylation-specific Polymerase Chain Reaction (PCR), pyrosequencing, Illumina arrays, and bisulfite sequencing, often produce inconsistent results. Blood collection, storage, and processing methods also significantly affect circulating biomarker measurements.

Established biomarkers pose stiff competition, making it hard for epigenetic markers to prove additional value. Tumor mutational burden, microsatellite instability, and PD-L1 expression already inform immunotherapy choices, while BRAF and NRAS mutations determine eligibility for targeted therapy. For epigenetic markers to be adopted in clinical practice, they must either outperform current tests or offer unique, complementary information that enhances outcomes—an ambitious goal that requires direct comparison studies.

Heterogeneity of melanoma—both within individual tumors and between different metastases in the same patient—creates difficulties for all biomarker strategies, but especially for epigenetic markers, which can vary widely across tumor regions. A single biopsy may not reflect the full tumor profile, although circulating biomarkers could potentially capture this heterogeneity by sampling multiple tumor sites. Researchers have employed multi-omics approaches, including single-cell techniques, to connect epigenetic alterations to melanoma pathogenesis. A comprehensive single-cell RNA-seq analysis across multiple cancer types, including melanoma, revealed shared gene expression patterns associated with stress and differentiation. These findings have enhanced our understanding of melanoma heterogeneity and the roles of various cell states in disease advancement [308].

Complete lack of cost-effectiveness data for melanoma epigenetic biomarkers is another obstacle. Healthcare systems require evidence that these tests improve outcomes to justify their expense. Without such data, insurers will not reimburse for the tests, and clinicians are unlikely to order them—even if they become available.

## 6 Conclusion

The review serves as a foundation for bringing to maturity the translational realities of epigenetic approaches to melanoma therapy. Recent progress in epigenetics offers hope for improving patient outcomes by enabling melanoma management to focus on personalized strategies that integrate both genetic and epigenetic data. As evidenced in this review, the epigenetic pathways that may impact melanoma initiation, progression, and overall virulence are diverse and complex. Therefore, therapeutic approaches will need to understand how epigenetic factors collaborate in aggregate to produce and perpetuate disease in individual patients. Utilizing AI to integrate data offers promise in this regard. While cancer remains a disease of the genome, epigenetic approaches offer novel strategies for correcting the consequences of an aberrant and mutated melanoma genome.

## Data Availability

Not applicable.

## Appendix A

**Table A1:** Aberrant DNA methylation genes in malignant melanoma

Predominant mechanism	Gene	Chromatin mark/Function	Expression	Reference
**Proliferation**	RASSF1A (Ras association domain family member 1, isoform A)	Promoter hypermethylation, Tumor suppressor	Down	[309]
	APC (Adenomatous Polyposis Coli)	Promoter1A hypermethylation, Tumor suppressor	Down	[310]
	KIT proto-oncogene, receptor tyrosine kinase (KIT)	KIT promoter hypermethylation in cutaneous MM; KIT exon mutation in mucosal, acral malignant melanoma (MM)	Down in cutaneous MM; Up in mucosal, acral MM	[311,312]
	RIPK3 (Receptor Interacting Serine/Threonine Protein Kinase 3)	Promoter hypermethylation, Tumor suppressor	Down	[313]
	SOCS3 (Suppressor of Cytokine Signaling 3)	Promoter hypermethylation, both tumor suppressive/promoting roles	Up or down	[314,315]
	TRIM16 (Tripartite motif containing 16)	Promoter hypermethylation, Tumor suppressor	Down	[316]
	MAPK13 (Mitogen-activated protein kinase 13)	Tumor suppressor. Hypermethylation in 67% of primary and 85% of metastatic melanomas.	Down	[317,318]
	RAR β (Retinoic acid receptor β)	Promoter hypermethylation, histone H3, H4 hypoacetylation	Down	[319,320]
	SPINT2 (Serine peptidase inhibitor Kunitz type 2)	Promoter hypermethylation, Tumor suppressive function by inhibition of a signal regulator of HGF (Hepatocyte growth factor)	Down	[321]
	LXN (Latexin)	Promoter hypermethylation, Tumor suppressor	Down	[322]
**Cell growth**	RASSF1A (Ras association domain family member 1, isoform A)	Promoter hypermethylation, Tumor suppressor	Down	[309]
	DPPIV (Dipeptidyl peptidase IV)	Promoter hypermethylation, Tumor suppressor	Down	[323]
	CDKN2A (Cyclin-dependent kinase inhibitor 2A)	Promoter hypermethylation, Tumor suppressor	Down	[324]
	TSPY (Testis-specific protein, Y-linked 1), CYBA (Cytochrome b-245 alpha chain), MTA2 (Metastasis associated 1 family member 2), MX1 (MX dynamin like GTPase 1), RPL37A (Ribosomal protein L37a), HSPB1 (Heat shock protein family B (small) member 1)	Promoter hypermethylation, Tumor suppressor	Down	[325,326]
	ESR1 (Estrogen Receptor 1)	Promoter hypermethylation, ERβ is the predominant ER in melanoma. ERβ has anticancer effect	Down with disease progression	[327,328]
	FES (Feline sarcoma oncogene)	Promoter hypermethylation, Tumor suppressor. Proto-oncogene.	Down	[40]
	SOCS1 (Suppressor of cytokine signaling 1)	Promoter hypermethylation, Tumor suppressor. More than 50% of melanomas	Down	[329,330]
	WFDC1 (WAP four-disulfide core domain 1)	Promoter hypermethylation, modulates inflammation	Down	[331]
**Migration**	RUNX3 (Runt-related transcription factor 3)	Promoter hypomethylation	Down	[332]
	ATF-3 (Activating Transcription Factor 3)	Promoter hypermethylation, Tumor suppressor	Down	[333]
	RASSF8 (Ras association domain family member 8	Promoter hypermethylation, Tumor suppressor	Down	[334]
	T/H Cadherin	Promoter hypermethylation, Tumor suppressor	Down	[335]
	TIMP3 (Tissue inhibitor of metalloproteinase-3)	Promoter hypermethylation, Tumor suppressor. Inhibits migration and invasion of melanoma cells	Down	[336]
**Invasion**	MITF (microphthalmia-associated transcription factor)	High MITF: differentiated, proliferative phenotype. Low MITF: dedifferentiated, invasive phenotype	Up or down	[337,338]
	FRZB (Frizzled-Related Protein)	Promoter hypermethylation, Tumor suppressor	Down	[339]
	MTAP (methylthioadenosine phosphorylase)	Promoter hypermethylation, Tumor suppressor. Melanoma cells re-expression of MTAP decreases invasive potential	Down	[340]
	E cadherin	Promoter hypermethylation, Tumor suppressor. Maintains cell-cell adhesion	Down	[341,342]
	T/H cadherin	Promoter hypermethylation, Tumor suppressor	Down	[343]
	SERPINB5 (Maspin)	Promoter hypermethylation, Tumor suppressor	Down	[344]
	GPX3 (glutathione peroxidase 3)	Promoter hypermethylation, Tumor suppressor	Down	[345]
	THBS1 (Thrombospondin-1)	Promoter hypermethylation, Tumor suppressor. Upregulates MMP 2,9 (matrix metalloproteinase 2,9)	Up	[346]
**Metastasis**	CDH11 (cadherin11)	Loss of tumor suppressor/loss of cell cycle (G0/G1 phase) arrest. Hypermethylation related to lymph node metastasis.	Up or down	[347]
	AGTR1 (Angiotensin II receptor type 1)	Promoter hypermethylation, Tumor suppressor	Down	[348]
	LOXL3 (lysyl oxidase-like 3)	Hypomethylation, oncogene Regulates phenotypic changes via the EMT transcription factors	Up	[349]
	PTEN (phosphatase and tensin homolog)	Promoter hypermethylation, Tumor suppressor	Down	[62]
	LINE-1 (Long Interspersed Nuclear Element-1)	Hypomethylation, genomic instability. Higher methylation level related with metastasis	Up or down	[350]
	RASSF6 (Ras association domain family member 6)	Promoter hypermethylation, Tumor suppressor	Down	[351]
	MMP-9	Intragenic hypermethylation. Higher MMP9 levels related to BRAFV600E mutation	Up	[352,353]
	SYNPO2 (synaptopodin-2)	Promoter hypermethylation, Tumor suppressor	Down	[354]
	TM (Thrombomodulin)	Promoter hypermethylation, Tumor suppressor. Binds to plasminogen and activates metalloproteases	Down	[355]
	TRIM16 (Tripartite motif containing 16)	Promoter hypermethylation, Tumor suppressor	Down	[356]
	TCF21 (Transcription factor 21)	Promoter hypermethylation, Tumor suppressor. Loss of TCF21 expression results in loss of Kindred Suppressor Sequence (KISS)1 expression, a metastasis suppressor	Down	[357]
	Tissue factor pathway inhibitor 2 (TFPI2)	Promoter hypermethylation, Tumor suppressor. High methylation subgroup related to worse prognosis	Down	[358]
	CLDN11 (Claudin 11)	Methylation 79% in skin metastases	Down	[359]
	SOCS1 (Suppressor of Cytokine Signaling 1)	Promoter hypermethylation, Tumor suppressor. Expression low in melanoma brain metastases	Up or down	[360]
	RASSF10 (Ras association domain family member 10)	Promoter hypermethylation, Tumor suppressor. 68% of primary melanoma and 91% of the metastatic melanoma	Down	[361]
	SyK (Spleen associated tyrosine kinase)	Promoter hypermethylation, Tumor suppressor. Non-Receptor-Associated Tyrosine Kinase	Down	[362]

**Table A2:** Clinical trials of epigenetic agents for melanoma treatment

Trial identifier	Study title	Phase	Epigenetic agent	Agent type	Status
NCT02608437	Guadecitabine in combination with Ipilimumab	Phase I	Guadecitabine	DNA methyltransferase 1 (DNMT1) inhibitor	Completed (long-term clinical benefits) [301,363]
NCT04250246	Guadecitabine with or without Ipilimumab plus Nivolumab	Phase II	Guadecitabine	DNMT1 inhibitor	Unknown status
NCT05089370	Oral Decitabine/Cedazuridine (DEC-C) in Combination with Nivolumab for Patients with Mucosal Melanoma	Phase I/II	Oral decitabine	DNMT1 inhibitor	Active, not recruiting
NCT00715793	Combination of Decitabine and Temozolomide in the Treatment of Patients with Metastatic Melanoma	Phase I/II	Decitabine	DNMT1 inhibitor	Completed
NCT01876641	Treatment of a Resistant Disease Using Decitabine Combined with Vemurafenib Plus Cobimetinib	Phase I	Decitabine	DNMT1 inhibitor	Terminated (failure to recruit)
NCT00925132	Treatment of Resistant Metastatic Melanoma Using Decitabine, Temozolomide and Panobinostat	Phase I/II	Decitabine, Panobinostat	DNMT1 inhibitor/Histone Deacetylase (HDAC) inhibitor	Terminated [364] (ineffective)
NCT01933061	Study to Determine Efficacy and Safety of CC-486 (oral azacitidine (AZA)) with Nab-Paclitaxel vs. Nab-Paclitaxel in Patients with Chemotherapy-Naïve Metastatic Melanoma	Phase II	Oral azacitidine	DNMT1 inhibitor	Withdrawn (sponsor decision)
NCT04648826	Aerosolized Azacytidine as Epigenetic Priming for Bintrafusp Alfa-Mediated Immune Checkpoint Blockade in Patients with Unresectable Pulmonary Metastases from Sarcomas, Germ Cell Tumors, or Epithelial Malignancies	Phase I/II	Aerosolized Azacytidine	DNMT1 inhibitor	Withdrawn (withdrawn before enrollment)
NCT02959437	Azacitidine Combined with Pembrolizumab and Epacadostat in Subjects with Advanced Solid Tumors (ECHO-206)	Phase I/II	Azacitidine/ Epacadostat	DNMT1 inhibitor/indoleamine 2,3-dioxygenase-1 (IDO1) inhibitor	Terminated (sponsor decision)
NCT00121225	Vorinostat in Treating Patients with Metastatic or Unresectable Melanoma	Phase II	Vorinostat	HDAC inhibitor	Completed [365]
NCT02836548	HDAC Inhibitor Vorinostat in Resistant BRAF V600 Mutated Advanced Melanoma	Phase I/II	Vorinostat	HDAC inhibitor	Completed [366]
NCT03022565	Vorinostat in Patients with Class 2 High Risk Uveal Melanoma	Phase I	Vorinostat	HDAC inhibitor	Withdrawn (withdrawn before enrollment)
NCT01587352	Vorinostat in Treating Patients with Metastatic Melanoma of the Eye	Phase II	Vorinostat	HDAC inhibitor	Active, not recruiting
NCT00667082	NPI-0052 and Vorinostat in Patients with Non-small Cell Lung Cancer, Pancreatic Cancer, Melanoma or Lymphoma	Phase I	Vorinostat	HDAC inhibitor	Completed
NCT01065467	Panobinostat (LBH589) in Patients with Metastatic Melanoma	Phase I	Panobinostat	HDAC inhibitor	Completed
NCT02032810	Histone Deacetylase (HDAC) Inhibitor Panobinostat with Ipilimumab in Patients with Unresectable III/IV Melanoma	Phase I	Panobinostat	HDAC inhibitor	Completed
NCT03982134	PDR001(spartalizumab) + Panobinostat for Melanoma and NSCLC	Phase Ib	Panobinostat	HDAC inhibitor	Withdrawn (withdrawn before enrollment)
NCT03590054	Abexinostat Pan-HDAC Melanoma, squamous cell carcinoma of head and neck, urothelial carcinoma, Non-small Cell Lung Cancer (NSCLC) In combination with Pembrolizumab	Phase I	Abexinostat (PanHDACi)	HDAC inhibitor	Completed (unpublished)
NCT02697630	Efficacy Study of Pembrolizumab with Entinostat to Treat Metastatic Melanoma of the Eye (PEMDAC)	Phase II	Entinostat	HDAC inhibitor	Completed (exhibits durable responses in a subset of patients) [367,368]
NCT00185302	Entinostat for Melanoma	Phase II	Entinostat	HDAC inhibitor	Completed (unpublished)
NCT02437136	Dose-Escalation Study of Entinostat with Pembrolizumab in NSCLC With Expansion Cohorts in NSCLC, Melanoma, and Colorectal Cancer (ENCORE-601)	Phase Ib/II	Entinostat + Pembrolizumab	HDAC inhibitor + anti-PD-1 (Programmed cell death protein-1)	Completed [278]
NCT03765229	Entinostat in combination with Pembrolizumab for Melanoma	Phase II	Entinostat	HDAC inhibitor	Completed (exhibits preliminary antitumor effects)
NCT03565406	Mocetinostat in Combination with Ipilimumab and Nivolumab for Melanoma	Phase I	Mocetinostat	HDAC inhibitor	Terminated (high response rate, high immune related toxicities) [369]
NCT04133948	Domatinostat/Nivolumab in combination with Nivolumab and Ipilimumab	Phase Ib	Domatinostat	LSD1 (Lysine-specific demethylase 1)/HDAC inhibitor	Active, not recruiting (domatinostat : no benefit to neoadjuvant anti-PD-1 ± anti-CTLA-4 [370]
NCT05170334	Binimetinib Plus Belinostat for Subjects with Metastatic Uveal Melanoma	Phase II	Belinostat (PanHDACi)	HDAC inhibitor	Recruiting
NCT03278665	4SC-202 in Combination with Pembrolizumab in Patients Primary Refractory/Non-responding to Prior AntiPD-1 Therapy (SENSITIZE)	Phase Ib/II	4SC-202 Domatinostat + Pembrolizumab	HDAC/LSD1 inhibitor + anti-PD-1	Completed (unpublished)
NCT04557956	Addition of Tazemetostat, to the Usual Treatment (Dabrafenib and Trametinib) for Metastatic Melanoma That Has Progressed on the Usual Treatment	Phase I/II	Tazemetostat + Dabrafenib + Trametinib	HDAC inhibitor + BRAF/MEK (B-Raf proto-oncogene, serine/threonine kinase/mitogen-activated extracellular signal-regulated kinase) inhibitor	Active, not recruiting
NCT03903458	Tinostamustine and Nivolumab in Advanced Melanoma	Phase I	Tinostamustine	alkylating deacetylase inhibitor (AK-DACi)	Unknown status
NCT03525795	CPI-1205 in combination with Ipilimumab for Melanoma, NSCLC, Renal cell carcinoma (RCC), Urothelial Carcinoma	Phase I	CPI-1205	EZH2 (enhancer of zeste 2) inhibitor	Completed (unpublished)
NCT02082977	GSK126 and Immunotherapy in Melanoma	Phase I	GSK126	EZH2 inhibitor	Terminated (modest anticancer activity at tolerable doses)
NCT05677373	PLX-2853 Uveal Melanoma in Combination with Trametinib	Phase I/II	PLX-2853	BRD4 (bromodomain-containing protein 4) inhibitor	Withdrawn (sponsor withdrawn support)
NCT04879017	FHD286 for Metastatic uveal melanoma	Phase I	FHD286	SMARCA4/2 (SWI/SNF-related, Matrix-associated, Actin-dependent Regulator of Chromatin A4/2) allosteric inhibitor	Terminated (business reasons)
NCT01829971	MRX34 (MiR-34a mimic) Liver cancer, Small cell lung cancer (SCLC), lymphoma, melanoma, MM, RCC, NSLCL	Phase I	MRX34	MiR-34a mimic	Terminated (serious immune related adverse events) [371,372]
NCT02862145	MRX34 with Dexamethasone premedication for Melanoma	Phase I/II	MRX34	MiR-34a mimic	Withdrawn (serious immune-related adverse events)
